# Hormonal Regulatory Patterns of LaKNOXs and LaBEL1 Transcription Factors Reveal Their Potential Role in Stem Bulblet Formation in LA Hybrid Lily

**DOI:** 10.3390/ijms222413502

**Published:** 2021-12-16

**Authors:** Yue Zhang, Zhen Zeng, Yubing Yong, Yingmin Lyu

**Affiliations:** 1 Beijing Key Laboratory of Ornamental Plants Germplasm Innovation & Molecular Breeding, China National Engineering Research Center for Floriculture, Beijing Laboratory of Urban and Rural Ecological Environment, College of Landscape Architecture, Beijing Forestry University, Beijing 100083, China; zhangyue@lfnu.edu.cn (Y.Z.); zengzh2020@163.com (Z.Z.); t20202505@csuft.edu.cn (Y.Y.); 2 Technical Innovation Center for Utilization of Edible and Medicinal Fungi in Hebei Province, Edible and Medicinal Fungi Research and Development Center of Hebei Universities, Langfang Normal University, Langfang 065000, China

**Keywords:** KNOX transcription factor, BELL transcription factor, interaction, phytohormone, bulblet formation, LA hybrid lily

## Abstract

In lily reproduction, the mechanism of formation of bulbs has been a hot topic. However, studies on stem bulblet formation are limited. Stem bulblets, formed in the leaf axils of under- and above-ground stems, provide lilies with a strong capacity for self-propagation. First, we showed that above-ground stem bulblets can be induced by spraying 100 mg/L 6-BA on the LA hybrid lily ‘Aladdin’, with reduced endogenous IAA and GA_4_ and a higher relative content of cytokinins. Then, expression patterns of three potential genes (two *KNOTTED1*-like homeobox (*KNOX*) and one partial *BEL1*-like homeobox (*BELL*)), during stem bulblet formation from our previous study, were determined by RT-qPCR, presenting a down-up trend in *KNOX*s and a rising tendency in *BELL*. The partial *BELL* gene was cloned by RACE from *L*. ‘Aladdin’ and denoted *LaBEL1*. Physical interactions of LaKNOX1-LaBEL1 and LaKNOX1-LaKNOX2 were confirmed by yeast two-hybrid and bimolecular fluorescence complementation assays. Furthermore, hormonal regulatory patterns of single LaKNOX1, LaKNOX2, LaBEL1, and their heterodimers, were revealed in transgenic *Arabidopsis*, suggesting that the massive mRNA accumulations of *LaKNOX1*, *LaKNOX2* and *LaBEL1* genes during stem bulblet formation could cause the dramatic relative increase of cytokinins and the decline of GAs and IAA. Taken together, a putative model was proposed that LaKNOX1 interacts with LaKNOX2 and LaBEL1 to regulate multiple phytohormones simultaneously for an appropriate hormonal homeostasis, which suggests their potential role in stem bulblet formation in *L*. ‘Aladdin’.

## 1. Introduction

Lily (*Lilium* spp.) is an important bulbous flower worldwide, with various flower types, rich colors and excellent ornamental effect. The bulb, as an important commercial and asexual-reproductive organ of lily, attracts much attention and its organogenesis mechanism has been a hot scientific topic. In recent years, the mechanism of in vitro bulblet formation has been thoroughly researched; however, knowledge about stem bulblet formation is extremely limited. Stem bulblets, formed in leaf axils of under- (bulblets) and above-ground stem (bulbils), can endow strong self-propagation ability to the lily [1,2,3].

Dynamic changes of multiple phytohormones have been demonstrated to be closely associated with new bulb formation in the lily. In transcriptome comparison of mother scales with or without new bulblets, hormone metabolism was one of the three main KEGG pathways (i.e., carbohydrate metabolism, hormone metabolism and photosynthesis) clustered by differentially expressed genes (DEGs) besides basic pathways [4]. Cytokinins (CKs) and gibberellins (GAs) have been shown to play antagonistic roles in new bulbil formation of *L. lancifolium* [1]. CKs not only stimulate the formation of new bulbs in the lily but also the formation of underground organs of other species [4,5,6], whereas GAs inhibit their formation [1,4,7]. In addition, auxin (IAA) is also considered to play an important role in new organ formation, with the highest number of DEGs involved in IAA signaling transduction during bulbil formation in *L. lancifolium* [1]. Wounding rapidly changed the distribution of endogenous auxin and created new auxin maximums on cutting scales at the paraxial side, resulting in asymmetric cell proliferation [8]. The initial IAA accumulation was also observed in the process of bulbil formation in *L. lancifolium* [1] and *L. sulphureum* [2]. However, after the initial increase, IAA level was then significantly inhibited [1]. The up-down trend of IAA was also observed during in vitro bulblet formation and underground-stem bulblet formation [3,9]. Thus, IAA is understood to have a double-effect on bulb formation [1], that is, IAA concentration first increases to promote meristem regeneration but then decreases to maintain appropriate hormone homeostasis in new meristem [10,11]. Increased endogenous ABA content has a positive correlation with bulblet formation [12]. Furthermore, an appropriate hormonal homeostasis seems to be crucial to organogenesis in plants [13]. In *Lycoris chinensis*, CKs/GAs and CKs/IAA were high when bulbils formed in two scales [14]. Similarly, ZR/GA_3_ and ZR/IAA reached a peak before new bulblets could be clearly seen on the axils of underground stems [3]. Multiple phytohormones are involved in the organogenesis of the lily bulb; therefore, it seems to be inefficient to only analyze a single hormone when investigating the molecular regulatory mechanism underlying stem bulblet formation. In the whole hormonal network, some key transcription factors (TFs) act as “hinges” which can simultaneously coordinate cross-reactions of multiple hormone-signaling pathways, such as KNOTTED1-like homeobox (KNOX) and BEL1-like homeobox (BELL) TFs.

Both KNOX and BELL TFs belong to the plant-specific three-amino acid loop extension (TALE) superclass which is characterized by a highly conserved DNA-binding region, homeodomain [15]. The KNOX family can be divided into two subclasses, class I and class II, based on sequence similarity and expression patterns [16]. Genetic analyses have implicated class I *KNOX* genes in alterations in cell fate, disruption of differentiation and, ultimately, new organ formation [17,18,19,20]. The BELL family is considered to have similar functions via interactions with class I KNOX [21,22]. Ectopic meristems formed near the veins of leaves in plants overexpressed the maize *KN1* gene [17]. Overexpressing *POTH1*, a *KNOX* homeobox gene, enhanced tuber formation in potato (*Solanum tuberosum*) [23]. *LcKNOX1* from *Lycoris chinensis* was found to be involved in axillary bud regeneration [14]. The expression of *AtqKNOX1* and *AtqKNOX2* in *Agave tequilana* was induced at bulbil initiation and increased during bulbil development [24]. In the lily, three class I *KNOX* genes, *LoATH1*, *LoSTM* and *LoKNAT6*, were reported to play a crucial role in both new meristem initiation and the regeneration capacity of explants during bulblet formation [8]. In potato, overexpressing *StBEL5* had positive effects on tuber formation [25]. 

Alteration in cell fates caused by ectopic expression of *KNOX* or *BELL* genes is accompanied by dynamic changes in phytohormone levels [13,26]. KNOX TFs regulate accumulation of CKs by promoting CK biosynthesis and signaling [13,27,28], while they reduce GA levels mainly due to the down-regulation of GA biosynthesis and up-regulation of GA degradation [25,29,30,31,32]. In addition, the antagonistic relationship between class I KNOX TFs and auxin is understood to promote organ initiation in plant development, and auxin is the main hormonal pathway regulated by KN1 [33,34]. Moreover, KNAT3 and BLH1 in *Arabidopsis* synergistically promoted the response to ABA [35]. However, the role of KNOX and BELL TFs in stem bulblet formation is unknown. 

In this study, we first determined that the formation of above-ground stem bulblets induced by 6-BA was accompanied by less endogenous IAA, GA_4_ and higher relative content of cytokinins. Then, RT-qPCR analysis revealed a down-up trend in *LaKNOX*s and a rising tendency in *LaBEL1*. Physical interactions between LaBEL1 and LaKNOX1, LaKNOX1 and LaKNOX2, were confirmed in vivo and *in vitro*. Ectopic expression in *Arabidopsis* and hormone detection by HPLC-MS uncovered the detailed regulatory patterns of single LaKNOX1, LaKNOX2, LaBEL1 TFs and their heterodimers. Based on this, we propose a putative regulatory mechanism of LaKNOX1, LaKNOX2 and LaBEL1 TFs in phytohormone homeostasis underlying stem bulblet formation in *L*. ‘Aladdin’.

## 2. Results

### 2.1. Effects of Exogenous 6-BA on Stem Bulblet Formation in L. ‘Aladdin’

Based on our previous experiments, it was found that underground stem bulblet formation in *L*. ‘Aladdin’ may be related to hormone alterations and three TALE superfamily transcription factors [3], which was further investigated in this study. Regarding hormonal changes, we previously found that the relative concentration of cytokinins peaked when bulblets were first observed in the axils of underground stems and suggested that high relative concentration of cytokinins is beneficial to bulblet formation [3]. Here, to further study cytokinin effects, the underground stem of *L*. ‘Aladdin’ was treated with 6-BA via root irrigation with distilled water as control. 

**Figure 1:**
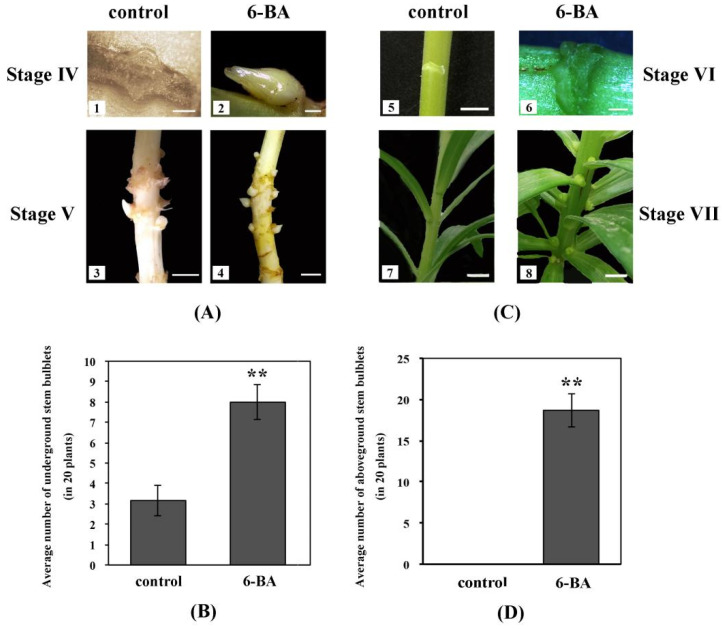
Effects of exogenous 6-BA on under- and above-ground stem bulblet formation in *L*. ‘Aladdin’. (**A**) shows that underground stem bulblet formation under 6-BA treatment was earlier than that in control. (**C**) shows exonenous 6-BA can induce aboveground stem bulblet formation in *L*. ‘Aladdin’. Scale bars for 1,2,6, 1 mm; for others, 1 cm. (**B**) shows the average number of underground stem bulblets under 6-BA treatment and control. (**D**) shows the average number of aboveground stem bulblets under 6-BA treatment and control. The bars show the mean ± SD from 20 plants. Asterisks show a significant difference ** *p* < 0.01 compared with the control.

Results showed that exogenous 6-BA clearly affected the formation time, quantity and quality of new bulblets under the ground (Figure 1 and Table 1). Regarding formation time, bulblets in the control condition were first observed at stage IV (70 days after cultivation) as in a previous study [3], while at this stage new bulblets under 6-BA treatment had grown (Figure 1A). After whole lily plants had completely withered, we randomly selected 20 plants to count the quantity of new bulblets per plant (Figure 1B) and found that the average number of bulblets with exogenous 6-BA was 8, clearly higher than 3.15 in control. Moreover, to evaluate the quality, new bulblets from 20 plants were divided into seven groups according to their diameter (Table 1). In the range above 1.8 cm, there were 6 bulblets under 6-BA treatment, accounting for 3.75%, which is higher than 1.59% in control (1 bulblet). In addition, bulblets of middle size (1.2–1.8 cm) under 6-BA accounted for 57.50%, slightly lower than 61.91% of control. As to bulblets less than 0.8 cm, the percentage increased from 7.94 (control) to 17.50 (6-BA). In sum, exogenous 6-BA application can not only accelerate bulblet formation, but significantly induce more bulblets in *L*. ‘Aladdin’ underground, especially in those of large (>1.8 cm) and small size (<0.8 cm).

In addition, we have previously suggested that all axils of the whole stem (both underground and aboveground) may have the potential to form bulblets when both outer environment and inner physiological conditions are appropriate, based on some special phenomena (Figure S1) [3]. In this study, 6-BA was also applied to aboveground stems to verify our previous assumption, since its promoting effects on underground stem bulblet formation have been confirmed. As anticipated, new bulblets were first observed in the middle and upper axils of aboveground stems after spraying 6-BA for 20 days (stage VI), whereas no bulblet were observed in control (Figure 1C). On average, 18.65 bulblets were formed on each plant (Figure 1D). We then divided all new bulblets into eight groups according to diameter (Table 2). Compared with underground bulblets, no large bulblet (>1.8 cm) was found. For the middle size (1.2–1.8 cm), there were 14 bulblets, accounting for 3.75%. Bulblets of small size (<0.8 cm) accounted for 73.99%. These results suggest that exogenous 6-BA application is needed for the formation of aboveground stem bulblets in *L*. ‘Aladdin’; however, the majority of such bulblets are small (<0.8 cm). 

Therefore, it has been confirmed that exogenous 6-BA application plays a crucial role in stem bulblet formation in *L*. ‘Aladdin’, but how endogenous hormone change occurs is still unknown.

### 2.2. Determination of Endogenous Hormone Content in Axils of Aboveground Stem after 6-BA Application

**Figure 2:**
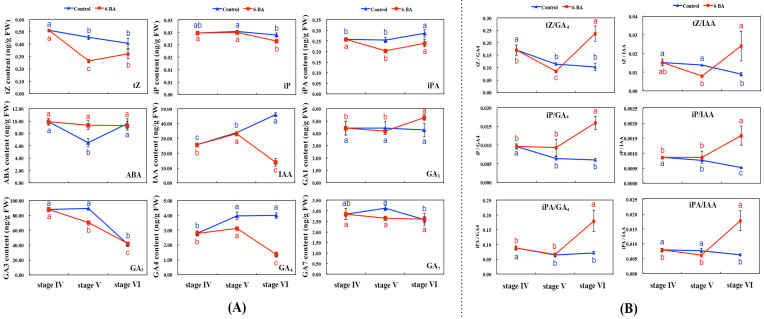
Endogenous hormone changes in the middle and upper axils of aboveground stems under 6-BA treatment from stage IV to stage VI by HPLC-MS. (**A**) shows the alterations of phytohormone levels in the process of aboveground stem bulblet formation in *L*. ‘Aladdin’. (**B**) shows the changes of relative concentrations of tZ, iP, and iPA compared with GA_4_ and IAA. The bars show the mean ± SD of three biological replicates.

HPLC-MS assay was utilized to analyze endogenous hormone changes in the middle and upper axils of aboveground stems under 6-BA treatment from stage IV to stage VI, contributing to key hormone identification during stem bulblet formation. It can be seen from Figure 2A that the application of exogenous 6-BA not only caused dynamic change in endogenous cytokinin (e.g., tZ, iP and iPA) concentrations, but also changes in endogenous gibberellin (e.g., GA_1_, GA_3_, GA_4_ and GA_7_), IAA and ABA concentrations. Most obviously, both IAA and GA_4_ concentrations decreased significantly from stage V to stage VI, opposite to that of control, suggesting IAA and GA_4_ play a more important role in stem bulblet formation than other hormones. Furthermore, the relative concentrations of three kinds of cytokinins (tZ, iP and iPA) were calculated, compared with IAA and GA_4_ (Figure 2B). Results showed that, at stage VI, when bulblets were first observed in leaf axils, the ratio of tZ/IAA, iP/IAA, iPA/IAA, tZ/GA_4_, iP/GA_4_ and iPA/GA_4_ under 6-BA treatment reached a peak which was noticeably higher than that of control. 

In sum, it has been shown that alterations of endogenous hormone concentrations directly affect stem bulblet formation in *L*. ‘Aladdin’, in particular, reducing IAA and GA_4_.

### 2.3. Expression Patterns of LaKNOX1, LaKNOX2 and LaBEL1 Genes during Stem Bulblet Natural Formation under the Ground

**Figure 3:**
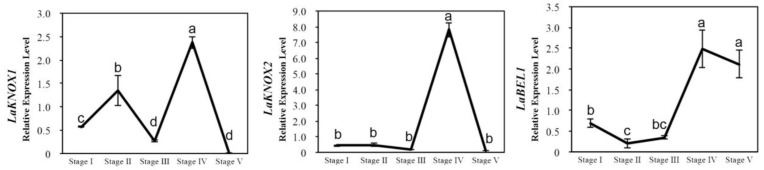
Expression patterns of *LaKNOX1*, *LaKNOX2*, and *LaBEL1* genes using RT-qPCR. Transcript changes of *LaKNOX1*, *LaKNOX2*, and *LaBEL1* genes in leaf axils of underground stem in the process of stem bulblet formation in *L*. ‘Aladdin’ (stage I, stage II, stage III, stage IV, and stage V). At stage V, underground stem bulblets can be clearly observed for the first time. The bars show the mean ± SD of three biological replicates.

It was speculated in our previous study that *LaKNOX1*, *LaKNOX2* genes and one *LaBELL* family gene might be involved in stem bulblet formation under the ground [3]. Here, we utilized RT-qPCR assay to analyze their expression patterns in leaf axils of underground stems from stage I to stage V, based on stage division in our previous study (For expression patterns of genes, see Figure 3; RT-qPCR primers are shown in Table S1) [3]. Results showed that such three genes were all significantly activated at stage IV, when new bulblets were first observed. Specifically, expression of two *LaKNOX* genes first decreased at stage III and then rebounded to a peak at stage IV, whereas one *LaBELL* family gene continued on an upward trend until it reached its highest point from stage III to stage IV. Unfortunately, we have been unable to determine what the changing tendencies of these three genes mean for stem bulblet formation in *L*. ‘Aladdin’ until now.

### 2.4. Cloning the Full Length of One LaBELL Family Gene from L. ‘Aladdin’

**Figure 4:**
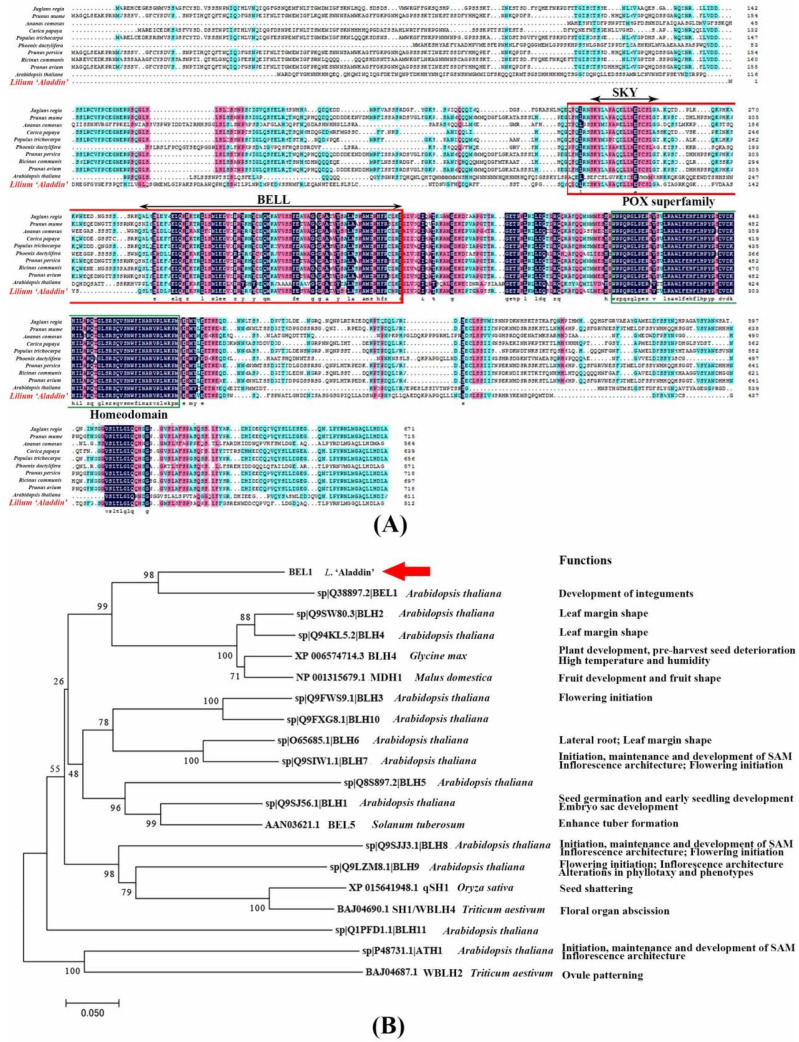
Characterization of LaBEL1 protein from *L*. ‘Aladdin’. (**A**) Alignment of deduced amino acid sequences of LaBEL1 with *Arabidopsis thaliana* AtBEL1, *Juglans regia* JrBEL1, *Prunus mume* PmBEL1, *Ananas comosus* AcBEL1, *Carica papaya* CpBEL1, *Populus trichocarpa* PtBEL1, *Phoenix dactylifera* PdBEL1, *Prunus persica* PpBEL1, *Ricinus communis* RcBEL1, and *Prunus avium* PaBEL1. Black arrowed lines indicate the location of the conserved SKY, BELL and homeodomain (green box). SKY and BELL domains compose the POX superfamily (red box). Identical amino acid residues are shaded in dark blue, similar in pink, and less similar in light blue. (**B**) Phylogenetic tree analysis of LaBEL1 with well-studied BELL proteins from other species. Accession numbers of other BELL proteins used in multiple sequence alignment and phylogenetic tree analysis are shown in Table S2.

It was necessary to characterize the specific functions of two *LaKNOX* genes and one *LaBELL* family gene isolated from *L*. ‘Aladdin’ to further investigate their potential role in stem bulblet formation. First, the full length of the *LaBELL* family gene (accession number MW882931) was cloned using RACE. This *BELL* homeobox gene contained a complete open reading frame (ORF) of 1539 bp, with 216 bp 5′ UTR and 135 bp 3′ UTR (primers for RACE are shown in Table S1). It encodes a putative protein of 512 aa with a calculated molecular mass of 56.9 kDa and a theoretical isoelectric point (PI) of 5.86. Multiple sequence alignment analysis confirms that it is a typical BELL homeobox protein with two conserved domains, namely POX superfamily (105–220 aa) in N-terminus and homeodomain (271–332 aa) in C-terminus (Figure 4A; NCBI accession numbers of other BELL proteins are shown in Table S2). POX superfamily consists of two conserved domains, SKY (109–127 aa) and BELL (146–218 aa). Moreover, a phylogenetic tree was constructed using the sequence of this putative BELL protein from *L*. ‘Aladdin’ and other well-studied BELLs (Figure 4B; NCBI accession numbers of other BELL proteins are shown in Table S2). Results showed that this novel BELL TF in lily was clustered closely to *Arabidopsis* BELL1 (AtBEL1); thus, it was designated LaBEL1. 

### 2.5. Subcellular Localization and Transactivation Assay of LaBEL1 and Two LaKNOX Proteins

**Figure 5:**
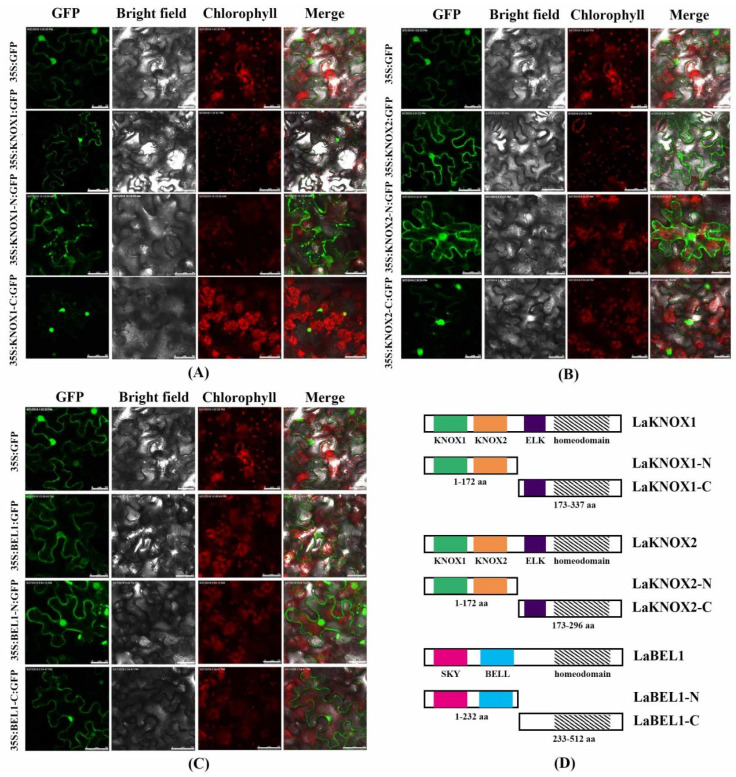
Subcellular localization of LaKNOX1 (**A**), LaKNOX2 (**B**), and LaBEL1 (**C**). 35S:GFP, 35S:LaKNOX1/LaKNOX2/LaBEL1:GFP, 35S:LaKNOX1-N/LaKNOX2-N/LaBEL1-N:GFP, and 35S:LaKNOX1-C/LaKNOX2-C/LaBEL1-C:GFP fusion proteins were transiently expressed in tobacco epidermal cells and observed by a laser scanning confocal microscope. GFP, bright field, chlorophyll, and merged images were taken (Scale bar for 35S:LaKNOX1:GFP, 75 μm; scale bars for 35S:LaKNOX1-C: GFP, 35S:LaKNOX2:GFP, and 35S:LaBEL1-C:GFP, 50 μm; scale bars for others, 25 μm). (**D**) The full-length of LaKNOX1, LaKNOX2, and LaBEL1 was split into N-terminal and C-terminal fragments based on the conserved domains.

To determine the potential regulatory mechanisms of LaKNOX1, LaKNOX2, and LaBEL1 TFs underlying bulblet formation in *L*. ‘Aladdin’, we analyzed their subcellular localization. Coding regions without a termination codon were fused to a GFP marker gene (primers for subcellular localization are shown in Table S1). These gene fusion constructs, and the GFP control in pBI121-GFP vector driven by CaMV35S promoter, were transiently expressed in the epidermis cells of tobacco (*Nicotiana benthamiana* L.). Furthermore, we split the full-length of LaKNOX1, LaKNOX2 and LaBEL1 into N-terminus and C-terminus, respectively, based on conserved domains to reveal their effects on subcellular localization (Figure 5D). The fluorescent proteins were visualized under a laser scanning confocal microscope (Figure 5). Results showed that LaKNOX1-GFP and LaKNOX2-GFP (full-length of LaKNOX1 and LaKNOX2) shared similar localization. Specifically, their fusion protein fluorescence signals were detected both in the nucleus and cytoplasm, as well as their N-terminal halves (containing the MEINOX domain) (Figure 5A,B). However, the fluorescence signals of C-terminus (ELK and homeodomain) were mainly detected in the nucleus. Using NLStradamus software, the NLS sequences ‘SKKKKKGNLPK’ were identified in the homeodomain of LaKNOX1 (239–249 aa) and ‘KEFMKKRKKGKLPK’ between ELK and homeodomain of LaKNOX2 (201–214 aa) (data not shown), which may explain why the fluorescence signals of C-terminal LaKNOXs were detected mainly in the nucleus. Thus, these results demonstrated that the N-termini of LaKNOX1 and LaKNOX2 TFs with the conserved MEINOX domain were responsible for the cytoplasmic localization, which may be the result of the disturbance of associated heterodimers via the MEINOX domain in the cytoplasm. Furthermore, LaBEL1-GFP and LaBEL1-N-GFP (N-terminal region) fusion proteins, and the fluorescence signal of LaBEL1-C-GFP (C-terminal region), without NLS sequence (data not shown), were also detected in both nucleus and cytoplasm (Figure 5C). Similar subcellular localization among LaKNOX1, LaKNOX2, and LaBEL1 proteins suggests the possibility of protein-protein interactions.

To detect transcriptional activity for the subsequent yeast two-hybrid (Y2H) experiment, the entire coding region, and N-terminal and C-terminal region coding sequences, of LaKNOX1, LaKNOX2 and LaBEL1, were inserted into the pGBDKT7 vector, containing the GAL4 DNA-binding domain (primers for transcriptional activity are shown in Table S1). These vectors and the pGBDKT7 (negative control) were then separately transformed into the yeast strain Y2HGold. The transactivation results indicated that all transformed yeast cells grew normally on SD/-Trp medium (Figure S2), indicating the successful transformation of fusion vectors. Yeast strains with full-length LaKNOX1, the N-terminus (LaKNOX1-N) and C-terminus (LaKNOX1-C), could not grow on the selection medium SD/-Trp-His/X-α-gal and SD/-His-Ade-Trp medium with 5 mM 3-amino-1,2,4-triazol (3-AT), suggesting that LaKNOX1, LaKNOX1-N and LaKNOX1-C are not transcriptional activators. As for LaKNOX2, it was interesting to find that the N-terminus (containing the MEINOX domain) could grow well on the SD/-His-Ade-Trp medium, appearing blue in the presence of α-galactosidase, whereas the full-length of LaKNOX2 could not. The results indicate that LaKNOX2 is not a transcriptional activator, although LaKNOX2-N can autonomously activate the reporter genes in Y2HGold in the absence of a prey protein. A similar phenomenon has also been identified in *Arabidopsis* BEL1 TF [36]. The simplest explanation for this discrepancy is that the LaKNOX2 protein may present a tertiary structure that masks the self-activation ability of its N terminus. Finally, LaBEL1 and LaBEL1-C were confirmed to have self-activation function, suggesting that the C terminus is responsible for the self-activation ability of LaBEL1. Therefore, the full-length LaKNOX1 and LaKNOX2, in addition to fragments without transactivation ability (LaKNOX1-N, LaKNOX1-C, LaKNOX2-C and LaBEL1-N), were chosen to conduct the following Y2H analysis.

### 2.6. LaKNOX1 Interacts with LaBEL1 and LaKNOX2

**Figure 6:**
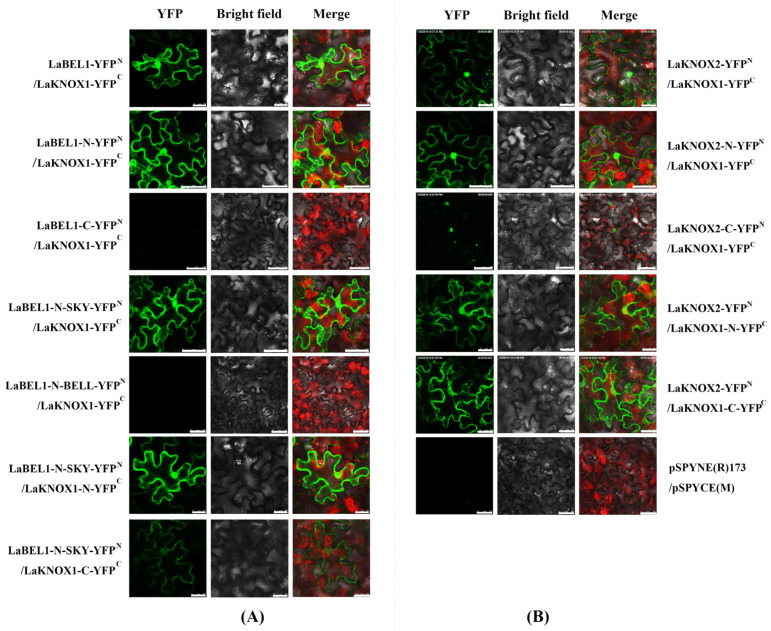
BiFC analysis using tobacco epidermal cells to study the interactions between LaKNOX1 and LaBEL1 (**A**), and LaKNOX1 and LaKNOX2 (**B**). Negative controls were pSPYNE(R)173 and pSPYCE(M). Red fluorescence signal in the merged picture represents chlorophyll. Scale bars for LaBEL1-N-YFPN/LaKNOX1-YFPC, LaBEL1-N-YFPN/LaKNOX1-YFPC, LaBEL1-C-YFPN/LaKNOX1-YFPC, LaBEL1-N-SKY-YFPN/LaKNOX1-N-YFPC, LaBEL1-N-BELL-YFPN/LaKNOX1-N-YFPC and pSPYNE(R)173/pSPYCE(M), 50 μm; for others, 25 μm.

**Figure 7:**
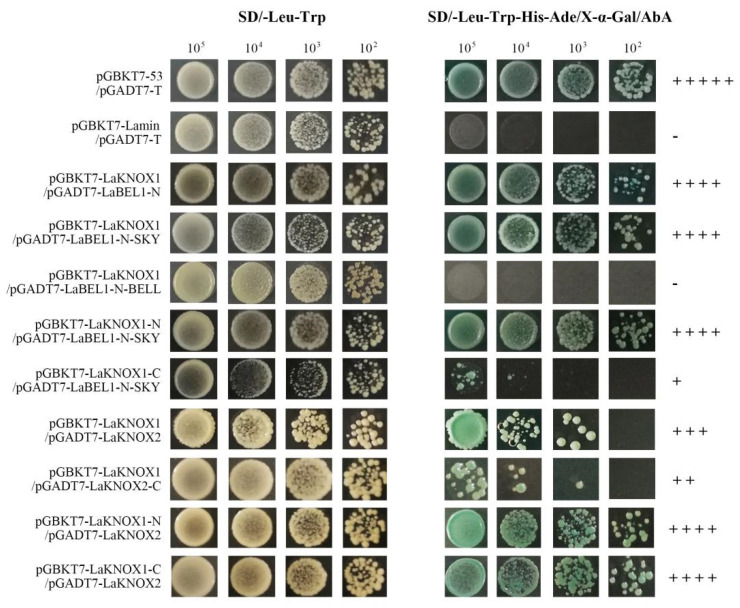
Y2H analysis to study the interaction among the full-length and fragments of LaKNOX1, LaKNOX2 and LaBEL1. Yeast co-transformants were grown on control medium SD/-Leu-Trp (**left panel**) and selective medium SD/-Leu-Trp-His-Ade/X-α-Gal/AbA (**right panel**). The pGBKT7-53/pGADT7-T and pGBKT7-Lamin/pGADT7-T were used as positive and negative controls, respectively. ‘+’ stands for interaction and ‘−’ indicates that there was no interaction observed. The number of plus signs means the ability to interact, that is, more plus signs means stronger interacting ability. The numbers on the top of picture, e.g., 10^5^, 10^4^, etc., represent the concentration of yeast liquid.

To investigate the interactions among LaKNOX1, LaKNOX2 and LaBEL1 proteins, bimolecular fluorescence complementation (BiFC) assays were first conducted in vivo (Figure 6; primers for BiFC are shown in Table S1). The candidate protein LaKNOX1 was fused to the pSPYCE(M) vector with C-terminal fragments of yellow fluorescent protein (YFP) (LaKNOX1-YFP^C^); meanwhile, LaKNOX2 and LaBEL1 were fused to the pSPYNE(R)173 vector with N-terminal fragments of YFP (LaKNOX2-YFP^N^ and LaBEL1-YFP^N^). Our results showed the co-expressions of LaBEL1-YFP^N^ and LaKNOX1-YFP^C^, and LaKNOX2-YFP^N^ and LaKNOX1-YFP^C^, generated strong fluorescence mainly in the nucleus and a little in the cytoplasm, while no YFP signal was detected in negative control pSPYNE(R)173/pSPYCE(M). Thus, LaKNOX1 could physically interact with LaBEL1 and LaKNOX2 both in the nucleus and cytoplasm. Moreover, the interaction between LaKNOX1 and LaKNOX2 was also verified by Y2H assay (Figure 7; primers for Y2H are shown in Table S1). 

Next, we sought to further determine whether LaBEL1-N and LaBEL1-C together or alone are involved in the interaction with LaKNOX1 (Figure 6 and Figure 7). LaBEL1-N showed strong interactive capability with LaKNOX1, which was similar to that of LaBEL1, while LaBEL1-C could not form a heterodimer with LaKNOX1. These results demonstrated that the N-terminus of LaBEL1 is responsible for the interaction with LaKNOX1. Moreover, each of two conserved domains in the N-terminal region of BELL TFs, SKY and BELL, has the potential to form an amphipathic α-helix that can mediate protein-protein interactions [37]. We then attempted to determine the effects on the interaction of SKY (109–127 aa, LaBEL1-N-SKY) and BELL (146–218 aa, LaBEL1-N-BELL) domains in the N-terminal region of LaBEL1 (Figure 6 and Figure 7). LaBEL1-N-SKY interacted with LaKNOX1 in the nucleus and cytoplasm, whereas LaBEL1-N-BELL did not, indicating that it was the SKY domain of LaBEL1 that mediated interaction with LaKNOX1. Additionally, we split LaKNOX1 into LaKNOX1-N (1–172 aa, containing MEINOX domain) and LaKNOX1-C (173–337 aa, containing ELK and homeodomain) to identify their impacts on the interaction (Figure 5D). Since the necessity of the SKY domain has been determined, the interactions between LaKNOX1-N or LaKNOX1-C and LaBEL1-N-SKY only were investigated here. LaKNOX1-N exhibited significantly stronger interactive capability to form a heterodimer with LaBEL1-N-SKY than LaKNOX1-C (Figure 6 and Figure 7). Therefore, both N- and C-terminal regions of LaKNOX1 protein are involved in the interaction, yet with unequal interactive capability. The MEINOX domain in the N-terminus mainly regulates the interaction with LaBEL1; however, there is also a weak interaction region lying in the C-terminal region of LaKNOX1.

To investigate the responsible domain(s) for the interaction between LaKNOX1 and LaKNOX2, we also split LaKNOX1 into LaKNOX1-N and LaKNOX1-C, and LaKNOX2 into LaKNOX2-N (1–172 aa, containing MEINOX domain) and LaKNOX2-C (173–296 aa, containing ELK and homeodomain) (Figure 5D). Results showed that the N- and C-terminal regions of LaKNOX1 and LaKNOX2 were all associated with the interaction. LaKNOX1 strongly interacts with LaKNOX2-N, but only weakly with LaKNOX2-C in the nucleus (Figure 6 and Figure 7). Moreover, LaKNOX2 showed similar interactive capability with both LaKNOX1-N and LaKNOX1-C, which was even stronger than that with the full-length of LaKNOX1 (Figure 7).

### 2.7. Altered Phytohormone Levels in Transgenic Arabidopsis Plants

**Figure 8:**
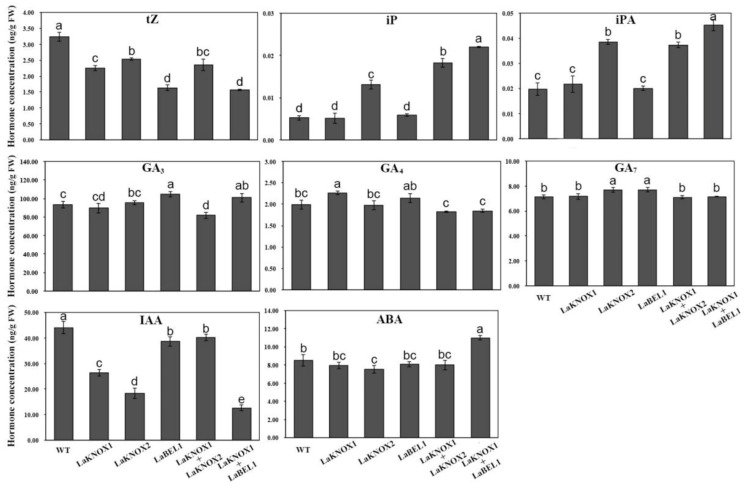
Endogenous hormone concentrations of WT, *LaKNOX1*, *LaKNOX2*, *LaBEL1*, *LaKNOX1 + LaKNOX2*, and *LaKNOX1 + LaBEL1* OE lines. The bars show the mean ± SD of three biological replicates. Different letters on the column denote statistically significant differences using Duncan’s multiple range test (*p* < 0.05).

To determine the specific regulatory patterns of LaKNOX1, LaKNOX2 and LaBEL1 TFs in phytohormone, we generated transgenic *Arabidopsis* plants overexpressing *LaKNOX1*, *LaKNOX2*, and *LaBEL1*, respectively, driven by the CaMV 35S promoter (primers used for overexpression vector construction are shown in Table S1; Figure S3). 12 independent transgenic positive lines (T_1_) (>20 transgenic lines) of each *LaKNOX1*, *LaKNOX2* and *LaBEL1* transgenic plants were assayed by RT-PCR to validate the transcript levels of introduced genes from *L*. ‘Aladdin’. Then, LaKNOX1-L7 and LaKNOX1-L10, LaKNOX2-L6 and LaKNOX2-L10, LaBEL1-L4 and LaBEL1-L8 with significantly high gene expression levels were selected for phytohormone concentration analyses (T_3_), after being verified by RT-qPCR. Furthermore, to compare the different effects on phytohormone between single TF and corresponding heterodimer, LaKNOX1 and LaKNOX2 were introduced together into *Arabidopsis* plants, as well as LaKNOX1 and LaBEL1. After selection on Murashige and Skoog (MS) medium containing 50 mg/L kanamycin (kan), T_0_-generation homozygous lines were extracted for gDNA and the presence of both *LaKNOX1* and *LaKNOX2*, or *LaKNOX1* and *LaBEL1* detected by PCR. Then, T_1_-generation homozygous lines were extracted and mRNA transcript levels were detected by RT-PCR. Finally, LaKNOX1 + LaKNOX2-L1 and LaKNOX1 + LaKNOX2-L5, and LaKNOX1 + LaBEL1-L1 and LaKNOX1 + LaBEL1-L9 were selected for subsequent analyses (T_3_). More importantly, to determine the detailed regulatory patterns of LaKNOX1, LaKNOX2 and LaBEL1 TFs and their heterodimers on phytohormones, HPLC-MS assay was utilized to assess three kinds of endogenous CKs (tZ, iP and iPA), three types of endogenous GAs (GA_3_, GA_4_ and GA_7_), IAA and ABA in whole adult plants of T_3_-generation transgenic *Arabidopsis* before bolting, in order to avoid interference in endogenous hormone content from different developmental stages of transgenic *Arabidopsis* (Figure 8). 

Regarding CKs, data analysis implicated two opposite functions of three TFs. The levels of iP and iPA increased in all transgenic lines whereas that of tZ decreased significantly compared with WT (*p* < 0.05), suggesting that LaKNOX1, LaKNOX2 and LaBEL1 TFs in lily can only raise isopentenyl-type CKs (iP and iPA) levels, not each type of CKs. Concerning the function of single TF, the promoting effect of LaKNOX2 on isopentenyl-type CK (iP and iPA) was stronger and the inhibiting impact on tZ was weaker than that of the other two TFs. More importantly, the concentrations of iP and iPA in *LaKNOX1* + *LaBEL1* transgenic plants were obviously higher than *LaKNOX1* or *LaBEL1* OE line, indicating the stronger promotional effect of LaKNOX1-LaBEL1 heterodimer on iP and iPA. Meanwhile, the tZ level in *LaKNOX1* + *LaBEL1* OE line was significantly lower than other lines (*p* < 0.05), except *LaBEL1*. These results suggest that LaKNOX1 could change the ratios among CK metabolites via interacting with LaBEL1 and LaKNOX2, up-regulating the level of isopentenyl-type CK, while down-regulating the tZ content. Moreover, regarding the GAs, GA_3_, GA_4_ and GA_7_ levels in *LaBEL1* OE line were significantly higher than that in *LaKNOX1* + *LaKNOX2* transgenic lines (*p* < 0.05), indicating that LaBEL1 TF probably plays a positive role in GAs concentrations in relation to the LaKNOX1-LaKNOX2 heterodimer. The concentrations of IAA and ABA in transgenic *Arabidopsis* plants were also detected. The results exhibited a significant decline of IAA levels in *LaKNOX1*, *LaKNOX2* and *LaKNOX1* + *LaBEL1* OE lines compared to WT (*p* < 0.05), suggesting that the LaKNOX1, LaKNOX2, LaKNOX1-LaBEL1 complex may negatively regulate IAA content. Interestingly, the negative control of LaKNOX1 or LaKNOX2 was weakened after forming the heterodimer. This suggested to us that LaKNOX1-LaKNOX2 heterodimer may have a totally different effect on IAA level compared with its single TF, which deserves further study in the future to specify their roles in the IAA metabolism pathway. As for ABA, it was clear that its concentration was up-regulated dramatically only in the *LaKNOX1* + *LaBEL1* double mutant (*p* < 0.05), suggesting the promoting impact of LaKNOX1-LaBEL1 heterodimer on ABA level. 

Furthermore, the relative concentrations of CKs (tZ, iP and iPA) compared with IAA, GA_3_, GA_4_ and GA_7_ were analyzed in different transgenic *Arabidopsis* lines (Figure S4). Results showed that the ratios of cytokinins to IAA in *LaKNOX2* and *LaKNOX1* + *LaBEL1* OE lines were much higher than for other transgenic lines and WT plants. Moreover, except for tZ/Gas, the ratios of iP or iPA to GA_3_, GA_4_ and GA_7_ in *LaKNOX2*, *LaKNOX1* + *LaKNOX2*, and *LaKNOX1* + *LaBEL1* OE lines were much higher than other transgenic lines and WT plants. In sum, LaKNOX2 TF alone had positive effects on the relative concentrations of CKs (tZ, iP and iPA) compared with IAA, and isopentenyl-type CKs (iP and iPA) with GAs; LaKNOX1 and LaBEL1 TFs were together able to raise the ratios of CKs/IAA and isopentenyl-type CKs/GAs; LaKNOX1-LaKNOX2 complex increased isopentenyl-type CKs/GAs ratios.

## 3. Discussion

In the lily, little is known about the regulatory mechanism underlying stem bulblet formation. In our previous study, the strong self-propagation capability of *L*. ‘Aladdin’ by production of underground stem bulblets was shown [3]. Thus, it represents ideal experimental material for investigation of bulb formation in the wild. Based on early-stage experiments, multiple hormones have been shown to be related to in vitro bulb formation [38,39]. An appropriate hormonal homeostasis appears to provide a general foundation for organogenesis [13]. The TALE TF superfamily (containing only the KNOX and BELL TF family in plants) has been reported to simultaneously modulate several phytohormones pathways, acting as ‘hinges’ in the hormonal network [40]. However, there has been no investigation of the functions of KNOX and BELL TFs, and their potential role in stem bulblet morphogenesis, in the lily. Based on our previous study, two class I *KNOX* genes, *LaKNOX1* and *LaKNOX2*, may be involved in underground stem bulblet formation with one partial *BELL* gene in *L*. ‘Aladdin’ [3]. Here, we further cloned the full-length of the partial *BELL* gene from *L*. ‘Aladdin’ and named it *LaBEL1* because it was clustered closely with *Arabidopsis* BELL1 in bioinformatic analysis. In *Arabidopsis*, *AtBELL1* is involved in lateral primordia and lateral integuments formation within developing seeds [36,37,41]. Thus, it is possible that BEL1 isolated from *L*. ‘Aladdin’ may share a similar function in regulating the formation of lateral organs.

Since the biological function of a protein is closely associated with its spatial localization [42], the subcellular localization analyses of the full length and conserved domains of LaBEL1, LaKNOX1 and LaKNOX2 proteins were first conducted. We observed that the full-length of LaBEL1, LaKNOX1 and LaKNOX2 localize both in the nucleus and cytoplasm, like AtMYC1 in *Arabidopsis* [43], and three R2R3-MYBs in *Salvia miltiorrhiza* [44]. In addition, their N-terminal fragments (containing a MEINOX domain in LaKNOXs and a POX domain in LaBEL1) have similar intracellular localization. It has been suggested that the subcellular localization of TF can be affected by other interacting TFs [43]. The MEINOX domain in LaKNOX-N and the POX domain in LaBEL1-N are highly conserved domains in both animals and plants, and have been shown to be involved in the heterodimerization between KNOX and BELL TFs [36,45]. After the interaction, there is a change in the subcellular localization of the heterodimers from the cytoplasm to the nucleus [46], implying that the location of full-length and N-terminal LaKNOXs and LaBEL1 TFs could be altered by other interacting proteins. Moreover, the C-terminal region (containing ELK and homeodomain) of LaKNOXs with NLS sequence mainly localize in the nucleus, consistent with the function of the homeodomain of binding to DNA and regulating downstream gene transcription [15]; however, the fluorescence signal of LaBEL1-C (containing only homeodomain) was detected both in the nucleus and the cytoplasm. This difference is assumed to be associated with the ELK domain which can function as a nuclear localization signal [47].

Heterodimerization is common in plants, providing a way to optimize the potential role of an individual TF in differentially modulating multiple downstream genes. To better explore the detailed functions of LaBEL1, LaKNOX1 and LaKNOX2 TFs, we then analyzed their interactions. LaKNOX1 was found to physically interact with LaBEL1, and with LaKNOX2 as well, using Y2H and BiFC assays. The fluorescence signal indicated that the LaKNOX1-LaBEL1 and LaKNOX1-LaKNOX2 heterodimers mainly localized in the nucleus and a little in the cytoplasm, suggesting that interactions also occurred in the cytoplasm. We suggest that heterodimers in the cytoplasm can be imported into the nucleus, based on similar findings in *Arabidopsis* [46]. Regarding the conserved domains responsible for interaction, the N-terminal region of LaBEL1 (containing the SKY and BELL domains) is assumed to be necessary, which is consistent with findings of AtBEL1 in *Arabidopsis* [36]. Furthermore, the SKY domain of LaBEL1 is responsible for interaction with LaKNOX1 while the BELL domain is not, even though, in theory, both the SKY and BELL domains have the potential to form an amphipathic α-helix that could mediate protein-protein interactions [37]. Moreover, both the N- and C-termini of LaKNOX TFs were found to mediate interaction. The involvement of the conserved MEINOX domain (N-terminus) in heterodimerization has been reported in many species [36,48,49]. In addition, we speculate that the involvement of the C-terminus (ELK domain and homeodomain) may be caused by the ELK domain.

Additionally, to gain further insights into the regulatory mechanisms underlying stem bulblet formation in *L*. ‘Aladdin’, exogenous 6-BA was applied by root-irrigation and spraying. The results indicated that it can accelerate and promote under- and above-ground stem bulblet formation. 6-BA is the first synthetic CK whose function is to promote cell division and proliferation. Nhut et al. found that the application of exogenous 6-BA in 1/2 MS medium was necessary for axillary pseudo-bulblets formation from in vitro shoot-tip-derived stem nodal segments of *L*. longiflorum, suggesting the important role of 6-BA in promoting bulblet formation in the axils of stems, which is consistent with our results [50]. Furthermore, regarding endogenous hormones, ZR, IAA, ABA, and GA_3_ have been shown to present dramatic shifts simultaneously during the organogenesis of underground stem bulblets in *L*. ‘Aladdin’ [3], as well as bulbil formation in *L*. lancifolium [1]. Thus, it is necessary to identify key regulators in the phytohormone network, which can modulate various hormones simultaneously. The KNOX and BELL TFs have already been shown to be involved in multiple hormone pathways in several species [35,36,51,52]. In this study, RT-qPCR analysis revealed that transcript changes of *LaKNOX1* and *LaKNOX2* genes first decreased and then increased during underground stem bulblet formation; meanwhile, expression of the *LaBEL1* gene was significantly induced. Unfortunately, we do not know how the changing trends in expression of the three genes affects concentrations of phytohormones in *L*. ‘Aladdin’.

So, our next goal was to determine the detailed regulatory patterns of LaKNOX1, LaKNOX2 and LaBEL1 TFs on phytohormone levels and the potential functions of transcript changes of *LaKNOX1*, *LaKNOX2* and *LaBEL1* genes during bulblet formation in *L*. ‘Aladdin’. At first, transgenic *Arabidopsis* plants, overexpressing *LaKNOX1*, *LaKNOX2* and *LaBEL1* genes, were generated to determine their effects on phytohormones. Moreover, as interactions between LaKNOX1 and LaKNOX2, LaKNOX1 and LaBEL1 had been confirmed, we also co-transformed *LaKNOX1* and *LaKNOX2* (*LaKNOX1 + LaKNOX2*), and *LaKNOX1* and *LaBEL1* (*LaKNOX1 + LaBEL1*) into *Arabidopsis* plants to determine whether there were differences in function between single TFs and their heterodimers. An HPLC-MS assay was utilized to detect the levels of endogenous CKs (tZ, iP and iPA), GAs (GA_3_, GA_4_ and GA_7_), IAA and ABA in transgenic lines. In other species, overexpressing *KNOX* or *BELL* genes can increase CK levels [13,23,53]. However, in this study, positive effects of KNOX TFs on CK levels were only found in isopentenyl-type CK (iP and iPA), not in tZ. Similar findings were also obtained from Frugis et al. [53]. iP and iPA levels were higher in *LaKNOX1 + LaBEL1* transgenic plants than in WT, *LaKNOX1* or *LaBEL1* OE lines, suggesting that LaKNOX1 TF is likely to significantly promote isopentenyl-type CK via interaction with LaBEL1. With respect to GAs, the effects of single TFs appeared to be complex, especially the positive regulation of LaBEL1 TF, which is inconsistent with previous studies [25,29,30,31,32]. To date, most studies have focused on the direct regulation of KNOX and BELL TFs on CK and GA levels. However, it is auxin that is the main hormonal pathway regulated by KN1 TF [33]. In this study, the IAA levels in *LaKNOX1*, *LaKNOX2*, and *LaKNOX1 + LaBEL1* lines were significantly lower than others, suggesting their negative control of IAA. Moreover, IAA content dramatically decreased in the *LaKNOX1 + LaBEL1* line whereas that in *LaKNOX1 + LaKNOX2* line was much higher than *LaKNOX1* or *LaKNOX2* OE lines. Thus, we speculate that LaKNOX1 TF may decrease the IAA level dramatically via interaction with LaBEL1, whereas the negative control could be weakened through formation of a heterodimer with LaKNOX2, which requires further study.

Finally, we sought to explain how the mRNA accumulations of *LaKNOX1*, *LaKNOX2* and *LaBEL1* genes modulate phytohormone concentration during stem bulblet formation. Prior to the emergence of bulblets, two *LaKNOX*s genes were substantially activated in axils of underground stems at stage IV. In maize (*Zea mays*), high expression of *KN1* is regarded as a switch to change cell fate from determinate to indeterminate to form new organs (small shoots) from fully differentiated tissues (leaves) [17]. Similarly, overexpression of *AtKNAT1* in lettuce results in a shift of leaf growth from determinate to shoot-like indeterminate [53]. Thus, it is possible that high expressions of *LaKNOX1* and *LaKNOX2* at stage IV may induce the transition of some cells in underground-stem axils from a differentiated to indeterminate state. In addition, the highest mRNA accumulation of *LaBEL1* also appeared at stage IV. Since heterodimerizations between LaKNOX1 and LaKNOX2, LaKNOX1 and LaBEL1 were investigated, we suggest that the three TFs function in the form of heterodimers from stage III to stage IV. We also noticed from Figure 3 that, from stage III to stage IV, mRNA accumulation of *LaKNOX2* was much more than that for the *LaKNOX1* and *LaBEL1* genes, suggesting that the LaKNOX2 TF is likely to function alone as well as interacting with LaKNOX1. Therefore, we speculate that the simultaneous overexpression of *LaKNOX1*, *LaKNOX2* and *LaBEL1* genes from stage III to stage IV may induce the rise of isopentenyl-type CKs (iP and iPA) and ABA levels, but a reduction in tZ, GA_3_, GA_4_ and IAA contents, based on hormone results from transgenic *Arabidopsis*. An accumulation of isopentenyl-type CKs, not the zeatin-type, was detected in the shift from determinate to indeterminate growth in lettuce leaf [53]. Tissue culture experiments have also indicated that using iP instead of 6-BA or TDZ in the medium can induce more bulbs [54,55]. In addition, increased endogenous ABA level is regarded as an important inducer of new bulb formation in lily [9,12,56]. The reduced tZ level was unexpected, but ZR content also decreased in bulbil formation of *L. lancifolium* [1]. Furthermore, isopentenyl-type CKs/GAs and CKs/IAA at stage IV may rise compared to that at stage III, based on transgenic *Arabidopsis* results. The high CK:low GA ratio has been shown to be essential for KNOX function to maintain the indeterminate state of cells and prevent cell differentiation [13]. Higher CKs/IAA was found to promote bulb formation in *Hyacinthus* and *Crocus* [57]. Therefore, increased expression of LaKNOX1, LaKNOX2 and LaBEL1 from stage III to stage IV may result in a change in cell fate from determinate to indeterminate to form new meristem, and eventually new bulblets.

## 4. Materials and Methods

### 4.1. Plant Materials and Exogenous Hormone Treatment

The three-year-old adult plants of LA (longiflorum × asiatic) hybrid cultivars ‘Aladdin’ with similar diameter selected in this study were planted into 1.5-L plastic pots after a two-month cool storage period (4 °C) in the greenhouse (116.3° E, 40.0° N) under controlled conditions. Based on our previous research, the process of bulblet formation in *L*. ‘Aladdin’ has been divided into five stages (Figure S5) [3]. Seedlings of *L*. ‘Aladdin’ were divided into four groups. Two groups were treated, respectively, by root-irrigation with 25 mg/pot 6-BA (CAISSON Labs, Smithfield, UT, USA) and water (as control) at stage I [58,59,60]. The other two groups were sprayed with 100 mg/L 6-BA (CAISSON Labs, Smithfield, UT, USA) and water (as control) at stage IV, when plant height was relatively stable and internodes of the upper stem were obvious. The second irrigation was repeated after 48 h. 

Wild-type *Arabidopsis thaliana* L. (Columbia background) was used for the transgenic experiment of *LaKNOX1*, *LaKNOX2*, *LaBEL1*, *LaKNOX1 + LaKNOX2* and *LaKNOX1 + LaBEL1*. WT and transformed plants were grown in an artificial climate chamber with a 16 h day-8 h night cycle at 16–22 °C and 70% relative humidity. Plants of *N. benthamiana* L. for subcellular localization and BiFC assays were cultured in the same conditions.

### 4.2. Effects of Exogenous 6-BA Application on Under- and Above-Ground Stem Bulblet Formation in L. ‘Aladdin’

Morphological alterations in under- and above-ground stem axils of L. ‘Aladdin’ were observed every 10 days from the first application of exogenous 6-BA at stage I and stage IV, respectively, to the appearance of early stem bulblets. Twenty plants were selected randomly in each group after their aboveground parts had withered completely to analyze the quality and quantity of stem bulblets.

### 4.3. Quantitative Real-Time Reverse Transcription-PCR Analysis

The division of stages in the process of underground-stem bulblet formation in *L*. ‘Aladdin’ has been described in detail in our previous study (stage I, stage II, stage III, stage IV and stage V) (Figure S5) [3]. For the expression patterns of *LaKNOX1*, *LaKNOX2* and *LaBEL1* during bulblet formation, axil samples of underground stem were collected at each stage and immediately frozen with liquid nitrogen and stored at −80°C for RNA isolation. 

Primers for RT-qPCR were designed in our previous study and are shown in Table S1 [3]. Quantitative real-time PCR reactions were performed using the Bio-Rad/CFX ConnectTM Real-Time PCR Detection System (Bio-Rad, San Diego, CA, USA) with SYBR^®^ Premix Ex TaqTM kit (Takara, Shiga, Japan). The relative mRNA expression levels were analyzed using the CFX Manager 3.0 Software and calculated using the 2^−ΔΔCT^ method with three technical replicates, using an internal reference gene encoding lily *tonoplast intrinsic protein 41* (*TIP41*) [61]. Three biological replicates were performed for each gene. 

### 4.4. Isolation and Sequencing of LaBEL1 from L. ‘Aladdin’

Total RNA was extracted from frozen inner scales of *L*. ‘Aladdin’ using the e.Z.N.A. Plant RNA Kit (OMEGA, Norcross, GA, USA) following the manufacturer’s protocols. The first-strand cDNA was synthesized from 2 μg total RNA using the PrimeScriptTM RT reagent Kit with gDNA Eraser (Takara, Shiga, Japan). A partial sequence of *BELL* gene was found in the transcriptome of *L*. ‘Aladdin’ apical meristems [62]. To obtain the full-length cDNA sequence, both 5′- RACE (rapid amplification of cDNA ends) and 3′- RACE were performed following the instruction of SMARTTM RACE cDNA Amplification Kit (Takara, Shiga, Japan) (Specific primers are shown in Table S1). The full-length cDNA sequence was then isolated from cDNA template of *L*. ‘Aladdin’ using LaBEL1-F/R listed in Table S1 and PrimeSTAR HS DNA Polymerase (Takara, Shiga, Japan). All PCR products were sub-cloned into pEASYT1-Blunt vector (TransGen Biotech, Inc., Beijing, China) and transfected into *Escherichia coli* Trans5α T1 competent cells (TransGen Biotech, Inc., Beijing, China). Selected clones were sequenced by the Beijing Ruibiotech Biotechnology Co., Ltd. (Beijing, China). After confirmation of sequencing, plasmid pEASYT1-LaBEL1 was used as a template for subsequent experiments. The theoretical molecular weight and isoelectric point were analyzed using the Expasy (https://www.expasy.org/resources/protparam accessed on 28 November) [63]. The homolog proteins of LaBEL1 were obtained through the BLAST (https://blast.ncbi.nlm.nih.gov/Blast.cgi accessed on 28 November) database. Multiple sequence alignment of amino acid sequences was carried out using DNAMAN 7.0 software. The phylogenetic tree was constructed using MEGA 5.0 software based on neighbor-joining method by bootstrap analyses with 1000 replicates [64]. NLStradamus software (www.moseslab.csb.utoronto.ca/NLStradamus/ accessed on 28 November) was used to predict nuclear localization signals (NLS). 

### 4.5. Subcellular Localization and Transactivation Assay 

For the subcellular localization assay, the whole coding regions of LaBEL1, LaKNOX1 and LaKNOX2 without the terminator codons were amplified using specific primers dwBEL1-*Xho*I and LaBEL1-*Sal*I, LaKNOX1-*Xho*I and LaKNOX1-*Sal*I, and LaKNOX2-*Xho*I and LaKNOX2-*Sal*I (Table S1). Furthermore, to identify the impact of different conserved domains on the subcellular localization, we split LaKNOX1, LaKNOX2 and LaBEL1 into N-terminus and C-terminus based on the conserved domain, LaKNOX1-N and LaKNOX1-C, LaKNOX2-N and LaKNOX2-C, LaBEL1-N and LaBEL1-C, respectively (Figure 5D). All PCR products were inserted into the *Xho*I and *Sal*I sites of vector pBI121-GFP using ClonExpress II One Step Cloning Kits (Vazyme, Piscataway, NJ, USA) to generate pBI121-BEL1-GFP, pBI121-BEL1-N-GFP, pBI121-BEL1-C-GFP, pBI121-KNOX1-GFP, pBI121-KNOX1-N-GFP, pBI121-KNOX1-C-GFP and pBI121-KNOX2-GFP, pBI121-KNOX2-N-GFP, pBI121-KNOX2-C-GFP fusion expression vectors. The recombinant plasmids and negative control (empty GFP vector) were transformed into *Agrobacterium tumefaciens* GV3101 and infiltrated into 4-week-old leaves of tobacco via an Agrobacterium-mediated transformation method [65]. After agro-infiltration for 32–48 h, the tobacco leaves were assayed for localization of the green fluorescence signal at 488 nm with a confocal laser scanning microscope (Leica TCS SP8, Leica Microsystems, Wetzlar, Germany) using a 500–530 nm emission filter. 

The transactivation and toxicity experiments were conducted according to the manual of Yeast Protocols Handbook (Takara, Shiga, Japan). The full-length coding region and truncated fragments of N-terminus and C-terminus as subcellular localization of LaKNOX1, LaKNOX2 and LaBEL1 were cloned with primers listed in Table S1. All PCR products were inserted into pGBKT7 vector between the *EcoR*I and *BamH*I restriction sites using ClonExpress II One Step Cloning Kits (Vazyme, Piscataway, NJ, USA) to generate fusion bait constructs as follows: pGBKT7-KNOX1 (1–337 aa), pGBKT7-KNOX1-N (1–172 aa, containing KNOX1 and KNOX2 domain), pGBKT7-KNOX1-C (173–337 aa, containing ELK and homeodomain), pGBKT7-KNOX2 (1–296 aa), pGBKT7-KNOX2-N (1–172 aa, containing KNOX1 and KNOX2 domain), pGBKT7-KNOX2-C (173–296 aa, containing ELK and homeodomain), pGBKT7-BEL1 (1–512 aa), pGBKT7-BEL1-N (1–232 aa, containing SKY and BELL domain), pGBKT7-BEL1-C (233–512 aa, containing homeodomain). Accuracy of the amplified fragments was checked by sequencing. Then all bait vectors and the pGBDKT7 (negative control) were separately transformed into the yeast strain Y2HGold using Quick & Easy Yeast Transformation Mix (Takara, Shiga, Japan). The transformed yeast cells were diluted 10-fold and 100-fold with sterile water, respectively, and then incubated on SD/-Trp, SD/-Trp-His, SD/-Trp-His/X-α-gal and SD/-His-Ade-Trp medium with 5 mM 3-AT under 30 °C for three days. The transactivation activity was detected according to their growth status and α-galactosidase activity. Three independent clones for each transformation were tested. 

### 4.6. Bimolecular Fluorescence Complementation (BiFC) Analysis

Vectors pSPYNE(R)173 and pSPYCE(M) were used to construct BiFC plasmids. To verify the interaction among LaKNOX1, LaKNOX2 and LaBEL1 in vivo, the full-length coding sequences of LaKNOX1 and LaKNOX2 were introduced into the vector pSPYCE(M) via *Spe*I/*Kpn*I, respectively, to generate C-terminal fusions with yellow fluorescent protein (YFP); the full-length coding sequence of LaBEL1 was subcloned via *BamH*I/*Kpn*I into the vector pSPYNE(R)173 to generate N-terminal fusions with YFP. Furthermore, we split LaKNOX1 and LaKNOX2 into N- and C-terminus inserted into the vector pSPYCE(M), and split LaBEL1 into LaBEL1-N, LaBEL1-C, LaBEL1-N-SKY, and LaBEL1-N-BELL, which were inserted into the vector pSPYNE(R)173. The primers used for BiFC are detailed in Table S1. 

For transient expression, *A. tumefaciens* strain GV3101 carrying the BiFC constructs was used for infiltration of 4-week-old leaves of tobacoo via *Agrobacterium*-mediated transformation method [65]. The *Agrobacterium* strains were infiltrated at an OD_600_ of 1.0 for the BiFC constructs. After 24 h of dark treatment and 36–48 h of LD treatment (16 h light), a GFP filter set was used for detection of pSPYNE(R)173 and pSPYCE(M) complexes and YFP fluorescence was excited at 514 nm with a confocal laser scanning microscope (Leica TCS SP8, Leica Microsystems, Wetzlar, Germany). 

### 4.7. Yeast Two-Hybrid (Y2H) Assay

The protein-protein interactions among two LaKNOX1, LaKNOX2 and LaBEL1 were investigated by conducting a yeast two-hybrid (Y2H) assay. A cDNA fragment encoding the N-terminal region (1–232 aa) of LaBEL1 (LaBEL1-N), deleting the auto-activation domain, was cloned into prey plasmid pGADT7 using the *EcoR*I and *BamH*I restriction sites so that it was in-frame with the GAL4 activation domain (AD). Furthermore, we spilt LaBEL1-N into two parts according to the conserved domains, LaBEL1-N-SKY (1–127 aa, containing SKY domain) and LaBEL1-N-BELL (136–232 aa, containing BELL domain), respectively, and inserted them into prey plasmid pGADT7. The coding sequences of LaKNOX1, LaKNOX1-N, LaKNOX1-C and LaKNOX2, LaKNOX2-C (which lacks the LaKNOX2-N for its autoactivation) were inserted into bait plasmid pGBKT7 to fuse with the DNA binding domain (BD) with the EcoRI and BamHI restriction sites. All primers are listed in Table S1. The bait vectors and prey vectors were co-transformed into *Saccharomyces cerevisiae* strain Y2HGold using Quick & Easy Yeast Transformation Mix (Takara, Shiga, Japan). Co-transformed yeast cells were selected on SD/-Leu-Trp plates and grown at 30 °C for 3–4 days. Next, we randomly selected six large single colonies and conducted colony PCR analysis to identify whether both bait and prey vectors successfully transformed into the yeast (data not shown). Positive single colonies were cultured overnight in SD/-Leu-Trp liquid medium (30 °C, 200 rpm). Yeast liquid was centrifuged with 4000 rpm for 3 min and the supernatant liquid was discarded. A quantity of 0.9% NaCl was added to suspend sedimentary yeast, and the previous operation was repeated. Afterwards, sufficient 0.9% NaCl was added to make the OD_600_ of the Y2HGold yeast liquid equal to 0.8. Finally, 10 μL of yeast liquid (1, 1/10, 1/100 and 1/1000) was cultured on SD/-Leu-Trp, SD/-Leu-Trp/X-α-Gal and SD/-Leu-Trp-His-Ade/X-α-Gal/AbA plates at 30 °C for 3–5 d. Three independent clones for each transformation were tested. Yeast cells transformed with pGBKT7-53 + pGADT7-T, or pGBKT7-Lam + pGADT7-T were used as positive or negative control, respectively.

### 4.8. Generation of Transgenic Arabidopsis 

The ORFs of *LaKNOX1*, *LaKNOX2*, and *LaBEL1* were amplified using gene-specific primers (Table S1) and cloned into a pBI121 binary vector, generating the overexpression constructs with the CaMV 35S promoter and the neomycin phosphotransferase II (nptII) selection gene. The recombinant plasmids were introduced into *A. tumefaciens* strain GV3101 and then transformed into wild-type *A. thaliana* ecotype Columbia-0 (Col-0) plants via the floral-dipping method [66]. Furthermore, inspired by the method of BiFC assay, we mixed the solution of *A. tumefaciens* strain GV3101 containing pBI121-LaKNOX1 plasmid and strain with pBI121-LaKNOX2 in the ratio of 1:1. Similarly, we blended the *A. tumefaciens* solution with pBI121-LaKNOX1 and that with pBI121-LaBEL1. The two kinds of mixed *A. tumefaciens* solution were used to generate co-transformed *Arabidopsis* using the floral-dip method [66]. Transformed seeds were treated at 4 °C in darkness for 48–72 h and then sowed on MS plates with 50 mg/L kan after sterilization. The plates were placed at 22 °C under a 12/12 (light/dark) photoperiod and constant white light (1000 lx) for seed germination. Resistant plants (T_0_) were transferred to soil and the existence of recombinant vectors was confirmed by PCR assay. T_3_-generation transgenic lines were selected to observe morphological changes in leaves and inflorescence, counting the rosette leaves and days from sowing to bolting to assess flowering time, and detect phytohormone levels.

### 4.9. Phytohormone Content Measurement

The middle and upper axils of aboveground stems under 6-BA treatment at stage IV, stage V and stage VI, and the whole adult plants of T_3_-generation transgenic *Arabidopsis* at time of flowering, were collected and used for measurement. Three biological replicates were performed for each sample. The levels of IAA, ABA, GA_3_, GA_4_, GA_7_, and tZ were detected using [^2^H_5_] IAA, [^2^H_6_] ABA, [^2^H_2_] GA_3_, [^2^H_2_] GA_4_ and [^2^H_2_] GA_7_, and [^2^H_5_] tZ (OlChemIm Ltd., Olomouc, Czech Republic) as internal standards, respectively. In addition, the external standards of iP and iPA (Sigma-Aldrich, St Louis, MO, USA) were employed for quantification. Endogenous hormones in transgenic plants were analyzed using high performance liquid chromatography-mass spectrometry (HPLC-MS) (QTRAP5500, AB Sciex, Redwood City, CA, USA). Their extraction and purification were performed using the method described previously [67]. Data processing was carried out by Analyst version 1.6.1 software. 

### 4.10. Statistical Analysis

The design of each experiment was as described above. The data for the average number and average weight of new bulblets on under- and above-ground stems, endogenous hormone levels in lily and transgenic *Arabidopsis*, and relative expression levels of *LaKNOX1*, *LaKNOX2* and *LaBEL1* were presented as mean values with standard errors. The statistical significance was determined using SPSS 22.0 (SPSS, Inc., Chicago, IL, USA) software. Letters denote statistically significant differences between the comparisons, based on Duncan’s multiple range test (*p* < 0.05).

## 5. Conclusions

**Figure 9:**
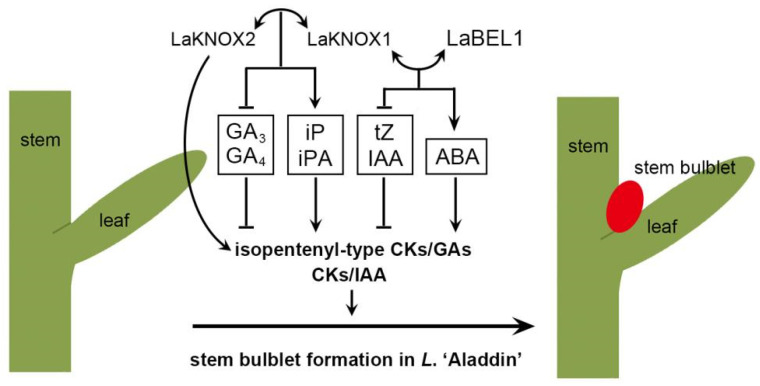
The putative regulatory mechanism of LaKNOX1, LaKNOX2 and LaBEL1 TFs in phytohormone underlying stem bulblet formation in *L*. ‘Aladdin’. ⟷ indicates the interaction, ↓ indicates promoting activity, and ⟂ indicates inhibiting activity.

In summary, a putative regulatory mechanism of LaKNOX1, LaKNOX2 and LaBEL1 TFs on stem bulblet formation in *L*. ‘Aladdin’ was proposed (Figure 9). From stage III to stage IV, LaKNOX1 interacts with LaKNOX2 to up-regulate isopentenyl-type CKs and down-regulate tZ, GA_3_ and GA_4_; heterodimerization between LaKNOX1 and LaBEL1 promotes isopentenyl-type CKs and ABA but inhibits tZ and IAA. In addition, LaKNOX2 TF alone increases the levels of isopentenyl-type CKs and GA_7_ but decreases that of tZ, IAA and ABA. In sum, the occurrence of LaKNOX1-LaKNOX2 and LaKNOX1-LaBEL1 heterodimers and LaKNOX2 TF alone could lead to increase of isopentenyl-type CKs/GAs and CKs/IAA, contributing to new bulblet formation. The results from this study provide insights into the potential role of LaKNOX1, LaKNOX2 and LaBEL1 TFs in underground-stem bulblet formation in *L*. ‘Aladdin’ by simultaneously modulating several kinds of phytohormones. Alterations of *LaKNOX1*, *LaKNOX2* and *LaBEL1* gene transcripts result in dynamic shifts of local balances of phytohormones and could affect bulblet formation in the lily.

## Figures and Tables

**Table 1:** Classification of underground stem bulblets from 20 plants in each group based on diameters.

Diameter, cm	6-BA			Control		
	Number	Average Weight ^1^, g	Percentage %	Number	Average Weight ^1^, g	Percentage %
>1.8	6	1.85 ± 0.31 a	3.75	1	2.56 a	1.59
1.6–1.8	13	1.41 ± 0.21 b	8.12	12	1.34 ± 0.22 b	19.05
1.4–1.6	35	1.00 ± 0.15 c	21.88	13	0.97 ± 0.14 c	20.63
1.2–1.4	44	0.68 ± 0.09 d	27.50	14	0.81 ± 0.11 cd	22.22
1.0–1.2	16	0.52 ± 0.12 de	10.00	9	0.59 ± 0.02 de	14.29
0.8–1.0	18	0.36 ± 0.12 ef	11.25	9	0.34 ± 0.01 ef	14.29
<0.8	28	0.17 ± 0.01 f	17.50	5	0.17 ± 0.01 f	7.93

^1^ Each value represents mean ± SD from 20 plants.

**Table 2:** Classification of aboveground stem bulblets from 20 plants in each group based on diameter.

Diameter, cm	6-BA		
	Number	Average Weight ^1^, g	Percentage, %
>1.8	0	0	0
1.6–1.8	4	1.25 ± 0.18 a	1.07
1.4–1.6	6	0.86 ± 0.12 b	1.61
1.2–1.4	4	0.54 ± 0.08 c	1.07
1.0–1.2	40	0.31 ± 0.02 d	10.72
0.8–1.0	43	0.21 ± 0.03 de	11.53
<0.8	276	0.08 ± 0.01 f	74

^1^ Each value represents mean ± SD from 20 plants.

## Supplementary Materials

The following are available online at https://www.mdpi.com/article/10.3390/ijms222413502/s1.
