# Supplementary material for: Hormonal Regulatory Patterns of LaKNOXs and LaBEL1 Transcription Factors Reveal Their Potential Role in Stem Bulblet Formation in LA Hybrid Lily
Source: Int J Mol Sci. 2021 Dec 16;22(24):13502. doi: 10.3390/ijms222413502 (PMC8703980; doi:10.3390/ijms222413502)
Supplement: Supplementary file 1 [file ijms-22-13502-s001.zip › ijms-1495516-SI.pdf]

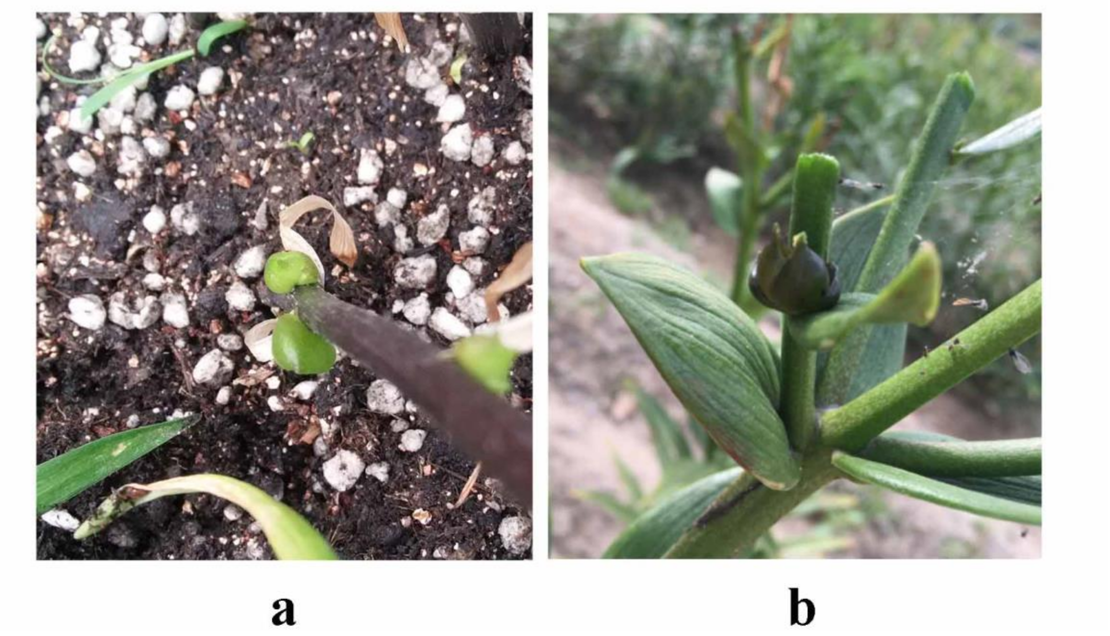

Figure S1. Special phenomena of stem bulblets above the ground in nature.

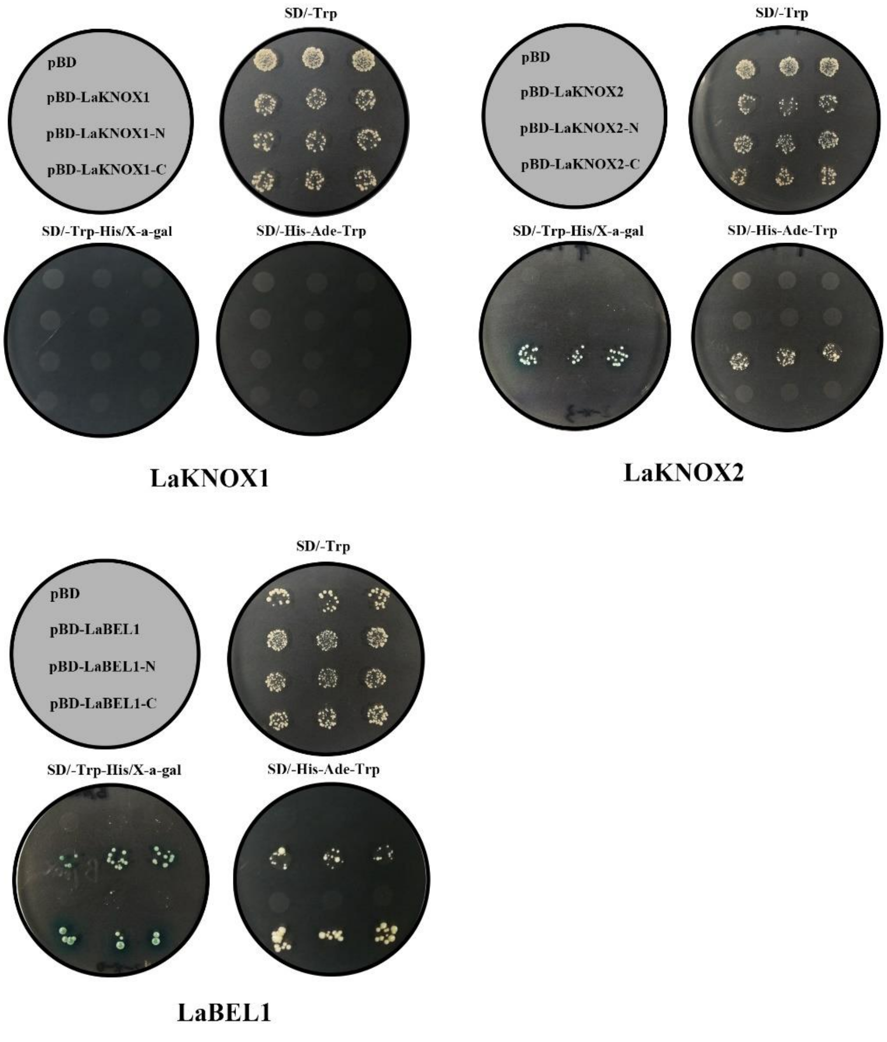

Figure S2. Transactivation assay of LaKNOX1, LaKNOX2, and LaBEL1 from *L.* 'Aladdin'.

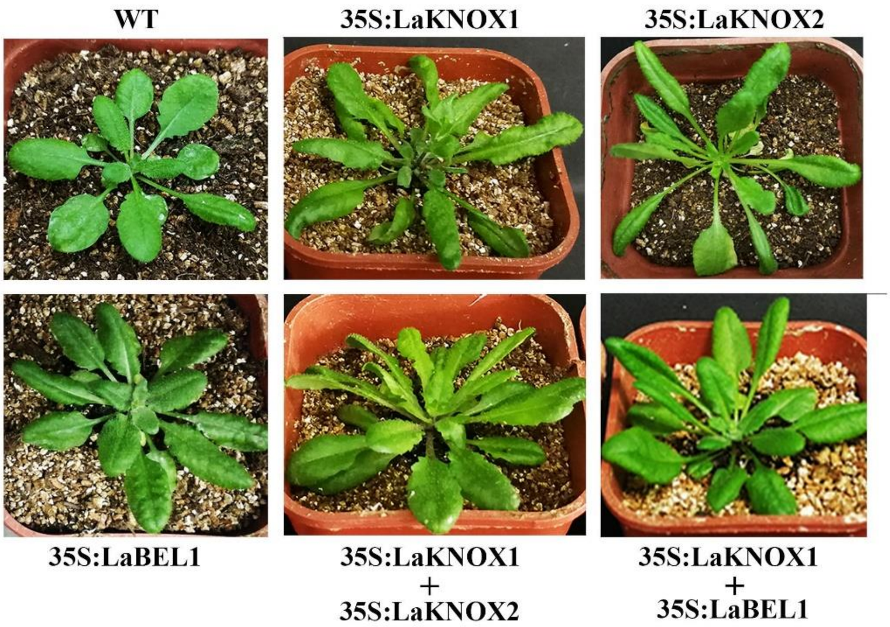

Figure S3. Transgenic Arabidopsis plants overexpressing *LaKNOX1*, *LaKNOX2*, and *LaBEL1* genes.

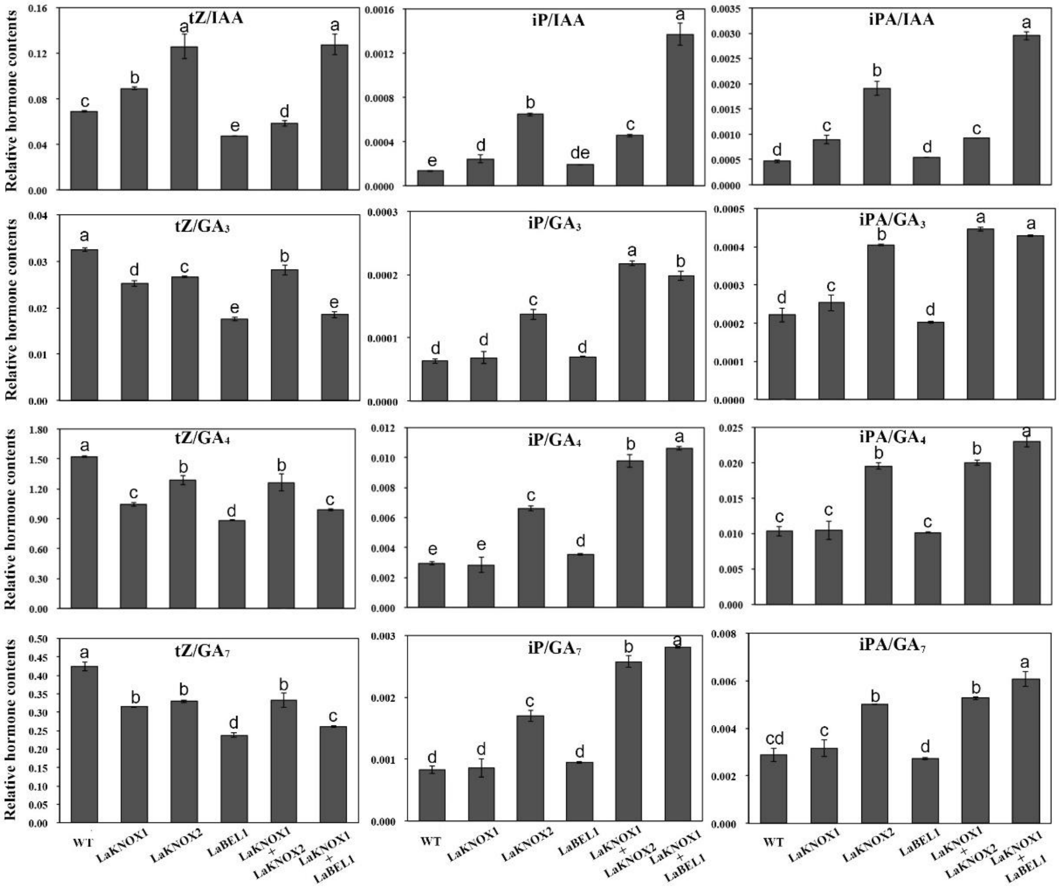

Figure S4. Relative hormone concentrations in transgenic Arabidopsis.

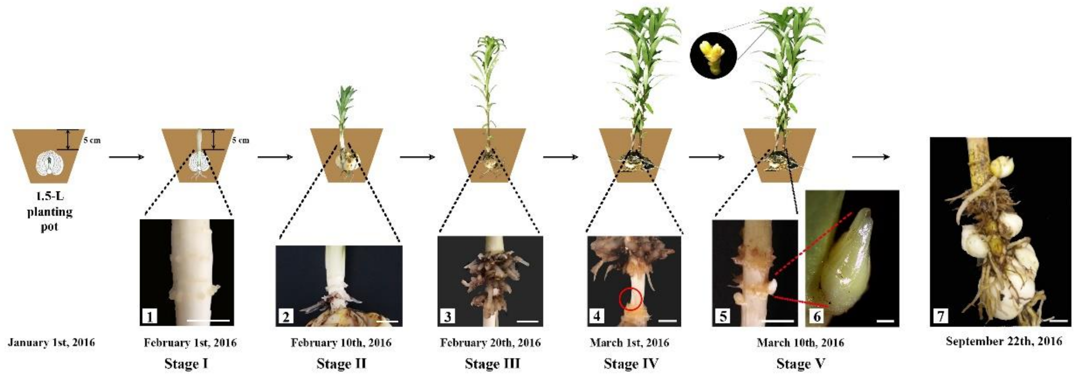

Figure S5. Division of stages in the process of underground stem bulblet formation in *L.* 'Aladdin'.

Table S1. Primers used in this study.

Primer name	Primer sequence (5'- 3')	Purpose
LaKNOX1-Y-F	AGAAGAAAGGCAATCTCCCAAA	Primers for RT-PCR and qRT-PCR assay of LaKNOX1
LaKNOX1-Y-R	GAAAGCCATCCATTACAACAAA	Primers for RT-PCR and qRT-PCR assay of LaKNOX1
LaKNOX2-Y-F	CTGCTACCGCTTACTCCGATGA	Primers for RT-PCR and qRT-PCR assay of LaKNOX2
LaKNOX2-Y-R	CCACCAGTCCAATAACGCCAAT	Primers for RT-PCR and qRT-PCR assay of LaKNOX2
LaBEL1-Y-F	CTTTTCGAGCACTTTCTTCAC	Primers for RT-PCR and qRT-PCR assay of LaBEL1
LaBEL1-Y-R	TTGGTTTCCTCCACATACATC	Primers for RT-PCR and qRT-PCR assay of LaBEL1
LaBEL1-M-F	TCCGGCGCAGGAGCTGTTGAAT	Amplification for the partial sequence of LaBEL1
LaBEL1-M-R	TCGACATCGCTTGGATACGGGT	Amplification for the partial sequence of LaBEL1
LaBEL1-3' GSP	AGGGAGCAGTAGGGGCGAAACACC	Primers for the LaBEL1 3' RACE analysis
LaBEL1-5' GSP	TATGACGCCGCATCTACGGGTTTACC	Primers for the LaBEL1 5' RACE analysis
UPM	GGGCAAGCAGTGGTATCAACGCAGAGT	RACE universal primer
LaBEL1-F	ATGGATCATGAAGGATTCGGGTATGGCG	Amplification for the cDNA sequences of LaBEL1
LaBEL1-R	CTACCCTGCAAGATCATGCAGCAGCTGA	Amplification for the cDNA sequences of LaBEL1
LaKNOX1-*Xho*I	CATTTACGAACGATACTCGAGATGGATGGCTTCACCCATCTCAGTG	Primers for subcellular localization of LaKNOX1
LaKNOX1-*Sal*I	CACCATCACTAGTACGTCGACCGGGCCCAGGCGGTATGTAC	Primers for subcellular localization of LaKNOX1
LaKNOX2-*Xho*I	CATTTACGAACGATACTCGAGATGGGGGACTTGTACAGTATACA	Primers for subcellular localization of LaKNOX2
LaKNOX2-*Sal*I	CACCATCACTAGTACGTCGACAGGACCAATTGTACCCGTATC	Primers for subcellular localization of LaKNOX2
LaBEL1-*Xho*I	CATTTACGAACGATACTCGAGATGGATCATGAAGGATTCGGGTATGGCG	Primers for subcellular localization of LaBEL1

LaBEL1-*Spe*I	CCCTTGCTCACCATCGTACTAGTCCCTGCAAGATCATGCAGCAGCTGA	Primers for subcellular localization of LaBEL1
LaKNOX1-N-*Xho*I	CATTTACGAACGATACTCGAGATGGATGGCTTCACCCATCTCAGTG	Primers for subcellular localization of LaKNOX1 N-terminus
LaKNOX1-N-*Sal*I	CACCATCACTAGTACGTCGACGCACAACGAGTTGAGCTGTGATTCG	Primers for subcellular localization of LaKNOX1 N-terminus
LaKNOX1-C-*Xho*I	CATTTACGAACGATACTCGAGTCTGATGGTGTTTCACCTCGCATCG	Primers for subcellular localization of LaKNOX1 C-terminus
LaKNOX1-C-*Sal*I	CACCATCACTAGTACGTCGACCGGGCCCAGGCGGTATGTAC	Primers for subcellular localization of LaKNOX1 C-terminus
LaKNOX2-N-*Xho*I	CATTTACGAACGATACTCGAGATGGGGGACTTGTACAGTATACA	Primers for subcellular localization of LaKNOX2 N-terminus
LaKNOX2-N-*Sal*I	CACCATCACTAGTACGTCGACAGTGCCAGATTCGTCAACATC	Primers for subcellular localization of LaKNOX2 N-terminus
LaKNOX2-C-*Xho*I	CATTTACGAACGATACTCGAGGATGTTGACGAATCTGGCACTT	Primers for subcellular localization of LaKNOX2 C-terminus
LaKNOX2-C-*Sal*I	CACCATCACTAGTACGTCGACAGGACCAATTGTACCCGTATC	Primers for subcellular localization of LaKNOX2 C-terminus
LaBEL1-N-*Xho*I	CATTTACGAACGATACTCGAGATGGATCATGAAGGATTCGGGTATGGCG	Primers for subcellular localization of LaBEL1 N-terminus
LaBEL1-N-*Sal*I	CACCATCACTAGTACGTCGACCTTCTCTCCCATAGCCTTTTTCGTTGCC	Primers for subcellular localization of LaBEL1 N-terminus
LaBEL1-C-*Xho*I	CATTTACGAACGATACTCGAGGATCCTACGGGAGCAGGGAGCAGTA	Primers for subcellular localization of LaBEL1 C-terminus
LaBEL1-C-*Sal*I	CACCATCACTAGTACGTCGACCCCTGCAAGATCATGCAGCAGCTGA	Primers for subcellular localization of LaBEL1 C-terminus
LaKNOX1-*Eco*RI	ATGGCCATGGAGGCCGAATTCATGGATGGCTTCACCCATCTCAGTG	Primers for transactivation analysis and Y2H to BD of LaKNOX1 full length
LaKNOX1-*Bam*HI	CCGCTGCAGGTCGACCGGATCCCGGGCCCAGGCGGTATGTAC	Primers for transactivation analysis and Y2H to BD of LaKNOX1 full length
LaKNOX2-*Eco*RI	ATGGCCATGGAGGCCGAATTCATGGGGGACTTGTACAGTATACA	Primers for transactivation analysis and Y2H to BD of LaKNOX2 full length
LaKNOX2-*Bam*HI	CCGCTGCAGGTCGACCGGATCCAGGACCAATTGTACCCGTATC	Primers for transactivation analysis and Y2H to BD of LaKNOX2 full length
LaBEL1-*Eco*RI	ATGGCCATGGAGGCCGAATTCATGGATCATGAAGGATTCGGGTATGGCG	Primers for transactivation analysis of LaBEL1 full length
LaBEL1-*Bam*HI	CCGCTGCAGGTCGACCGGATCCCCCTGCAAGATCATGCAGCAGCTGA	Primers for transactivation analysis of LaBEL1 full length
LaKNOX1-N-*Eco*RI	ATGGCCATGGAGGCCGAATTCATGGATGGCTTCACCCATCTCAGTG	Primers for transactivation analysis and Y2H to BD of LaKNOX1 N-terminus
LaKNOX1-N-*Bam*HI	CCGCTGCAGGTCGACCGGATCCGCACAACGAGTTGAGCTGTGATTCG	Primers for transactivation analysis and Y2H to BD of LaKNOX1 N-terminus
LaKNOX1-C-*Eco*RI	ATGGCCATGGAGGCCGAATTCTCTGATGGTGTTTCACCTCGCATCG	Primers for transactivation analysis and Y2H to BD of LaKNOX1 C-terminus

LaKNOX1-C-*Bam*HI	CCGCTGCAGGTCGACCGGATCCCGGGCCCAGGCGGTATGTAC	Primers for transactivation analysis and Y2H to BD of LaKNOX1 C-terminus
LaKNOX2-N-*Eco*RI	ATGGCCATGGAGGCCGAATTCATGGGGGACTTGTACAGTATACA	Primers for transactivation analysis of LaKNOX2 N-terminus
LaKNOX2-N-*Bam*HI	CCGCTGCAGGTCGACCGGATCCAGTGCCAGATTCGTCAACATC	Primers for transactivation analysis of LaKNOX2 N-terminus
LaKNOX2-C-*Eco*RI	ATGGCCATGGAGGCCGAATTCGATGTTGACGAATCTGGCACTT	Primers for transactivation analysis and Y2H to BD of LaKNOX2 C-terminus
LaKNOX2-C-*Bam*HI	CCGCTGCAGGTCGACCGGATCCAGGACCAATTGTACCCGTATC	Primers for transactivation analysis and Y2H to BD of LaKNOX2 C-terminus
LaBEL1-N-*Eco*RI	ATGGCCATGGAGGCCGAATTCATGGATCATGAAGGATTCGGGTATGGCG	Primers for transactivation analysis of LaBEL1 N-terminus
LaBEL1-N-*Bam*HI	CCGCTGCAGGTCGACCGGATCCCTTCTCTCCCATAGCCTTTTTCGTTGCC	Primers for transactivation analysis of LaBEL1 N-terminus
LaBEL1-C-*Eco*RI	ATGGCCATGGAGGCCGAATTCGATCCTACGGGAGCAGGGAGCAGTA	Primers for transactivation analysis of LaBEL1 C-terminus
LaBEL1-C-*Bam*HI	CCGCTGCAGGTCGACCGGATCCCCCTGCAAGATCATGCAGCAGCTGA	Primers for transactivation analysis of LaBEL1 C-terminus
LaKNOX2-AD-*Eco*RI	GCCATGGAGGCCAGTGAATTCATGGGGGACTTGTACAGTATACA	Primers for Y2H to AD of LaKNOX2 full length
LaKNOX2-AD-*Bam*HI	CAGCTCGAGCTCGATGGATCCAGGACCAATTGTACCCGTATC	Primers for Y2H to AD of LaKNOX2 full length
LaKNOX2-C-AD-*Eco*RI	GCCATGGAGGCCAGTGAATTCGATGTTGACGAATCTGGCACTT	Primers for Y2H to AD of LaKNOX2 C-terminus
LaKNOX2-C-AD-*Bam*HI	CAGCTCGAGCTCGATGGATCCGAGGACCAATTGTACCCGTATC	Primers for Y2H to AD of LaKNOX2 C-terminus
LaBEL1-N-AD-*Eco*RI	GCCATGGAGGCCAGTGAATTCATGGATCATGAAGGATTCGGGTATGGCG	Primers for Y2H to AD of LaBEL1 N-terminus
LaBEL1-N-AD-*Bam*HI	CAGCTCGAGCTCGATGGATCCGCTTCTCTCCCATAGCCTTTTTCGTTGCC	Primers for Y2H to AD of LaBEL1 N-terminus
LaBEL1-N-SKY-AD-*Bam*HI	CAGCTCGAGCTCGATGGATCCGGGCACCCAGACTGCAGAATTCATTCAAC	Primers for Y2H to AD of SKY domain in the N-terminal region of LaBEL1
LaBEL1-N-BELL-AD-*Eco*RI	GCCATGGAGGCCAGTGAATTCTAAACCCGTAGATGCGGCGTCGTACAGT	Primers for Y2H to AD of BELL domain in the N-terminal region of LaBEL1
LaBEL1-YNE-*Bam*HI	TGGCGCGCCACTAGTGGATCCATGGATCATGAAGGATTCGGGTATGGCG	Primers for BiFC of LaBEL1 full length
LaBEL1-YNE-*Kpn*I	CTCCATCCCGGGAGCGGTACCCCCTGCAAGATCATGCAGCAGCTGA	Primers for BiFC of LaBEL1 full length
LaBEL1-N-YNE-*Kpn*I	CTCCATCCCGGGAGCGGTACCCTTCTCTCCCATAGCCTTTTTCGTTGCC	Primers for BiFC of LaBEL1 N-terminus
LaBEL1-N-SKY-YNE-*Bam*HI	TGGCGCGCCACTAGTGGATCCATGGATCATGAAGGATTCGGGTATGGCG	Primers for BiFC of SKY domain in the N-terminal region of LaBEL1

LaBEL1-N-SKY-YNE-*Kpn*I	CTCCATCCCGGGAGCGGTACCGGCACCCAGACTG-CAGAATTCATTCAAC	Primers for BiFC of SKY domain in the N-terminal region of LaBEL1
LaBEL1-N-BELL-YNE-*BamH*I	TGGCGCGCCACTAGTGGATCCTAAACCCGTAGATGCGGCGTCG-TACAGT	Primers for BiFC of BELL domain in the N-terminal region of LaBEL1
LaBEL1-N-BELL-YNE-*Kpn*I	CTCCATCCCGGGAGCGGTACCCTTCTCTCCCATAGCCTTTTTCGTTGCC	Primers for BiFC of BELL domain in the N-terminal region of LaBEL1
LaBEL1-C-YNE-*Bam*HI	TGGCGCGCCACTAGTGGATCCGATCCTACGGGAGCAGGGAGCAGTA	Primers for BiFC of LaBEL1 C-terminus
LaKNOX1-YCE-*Spe*I	GCTGGGCCCAGGCCTACTAGTATGGATGGCTTCACCCATCTCAGTG	Primers for BiFC of LaKNOX1 full length
LaKNOX1-YCE-*Kpn*I	CTCCTACCCGGGAGCGGTACCCGGGCCCAGGCGGTATGTAC	Primers for BiFC of LaKNOX1 full length
LaKNOX1-N-YCE-*Kpn*I	CTCCTACCCGGGAGCGGTACCGCACAACGAGTTGAGCTGTGATTCG	Primers for BiFC of LaKNOX1 N-terminus
LaKNOX1-C-YCE-*Spe*I	GCTGGGCCCAGGCCTACTAGTTCTGATGGTGTTTCACCTCGCATCG	Primers for BiFC of LaKNOX1 C-terminus
LaKNOX2-YCE-*Spe*I	GCTGGGCCCAGGCCTACTAGTATGGGGGACTTGTACAGTATACA	Primers for BiFC of LaKNOX2 full length
LaKNOX2-YCE-*Kpn*I	CTCCTACCCGGGAGCGGTACCAGGACCAATTGTACCCGTATC	Primers for BiFC of LaKNOX2 full length
LaKNOX2-YNE-*BamH*I	TGGCGCGCCACTAGTGGATCCATGGGGGACTTGTACAGTATACA	Primers for BiFC of LaKNOX2 full length
LaK-NOX2-YNE-*Kpn*I	CTCCATCCCGGGAGCGGTACCAGGACCAATTGTACCCGTATC	Primers for BiFC of LaKNOX2 full length
LaKNOX2-N-YCE-*Kpn*I	CTCCTACCCGGGAGCGGTACCAGTGCCAGATTCGTCAACATC	Primers for BiFC of LaKNOX2 N-terminus
LaKNOX2-C-YCE-*Spe*I	GCTGGGCCCAGGCCTACTAGTGATGTTGACGAATCTGGCACTT	Primers for BiFC of LaKNOX2 C-terminus
LaKNOX2-N-YNE-*Kpn*I	CTCCATCCCGGGAGCGGTACCAGTGCCAGATTCGTCAACATC	Primers for BiFC of LaKNOX2 N-terminus
LaKNOX2-C-YNE-*Bam*HI	TGGCGCGCCACTAGTGGATCCGATGTTGACGAATCTGGCACTT	Primers for BiFC of LaKNOX2 C-terminus
LaKNOX1-*BamH*I	GGATCCATGGATGGCTTCACCCATCTC	Primers for overexpression vector construction
LaKNOX1-*EcoR*I	GAATTCTCACGGGCCCAGGCGGTATGT	Primers for overexpression vector construction
LaKNOX2-*Xba*I	TCTAGAATGGGGGACTTGTACAG	Primers for overexpression vector construction
LaKNOX2-*Sac*I	GAGCTCTCAAGGACCAATTGTACC	Primers for overexpression vector construction

LaBEL1-*Xba*I	TCTAGAATGGATCATGAAGGATTCGGGTATGGCG	Primers for overexpression vector construction
LaBEL1-*Sma*I	CCCGGGTACCCTGCAAGATCATGCAGCAGCTGA	Primers for overexpression vector construction
35S	GACGCACAATCCCACTATC	Universal primer
AtACTIN2-F	TCCTCTTAACCCAAAGGCCAACAGA	Reference gene in Arabidopsis
AtACTIN2-R	TGAGACACACCATCACCAGAATCCA	Reference gene in Arabidopsis

Single underlines are enzyme recognition site.

Table S2. NCBI accession numbers of other BELL proteins used in multiple sequence alignment and phylogenetic tree analysis.

Name	Species	NCBI Accession Number
JrBEL1	*Juglans regia*	XP_018852535.1
PmBEL1	*Prunus mume*	XP_008238940.1
AcBEL	*Ananas comosus*	OAY81307.1
CpBEL1	*Carica papaya*	XP_021887927.1
PtBEL1	*Populus trichocarpa*	XP_024447318.1
PdBEL1	*Phoenix dactylifera*	XP_008794768.1
PpBEL1	*Prunus persica*	XP_007210325.1
RcBEL1	*Ricinus communis*	XP_015571681.1
PaBEL1	*Prunus avium*	XP_021820869.1
AtBEL1	*Arabidopsis thaliana*	sp|Q38897.2|
AtATH1	*Arabidopsis thaliana*	sp|P48731.1|
AtBEL1	*Arabidopsis thaliana*	sp|Q38897.2|
AtBLH1	*Arabidopsis thaliana*	sp|Q9SJ56.1|
AtBLH2/SAW1	*Arabidopsis thaliana*	sp|Q9SW80.3|
AtBLH3	*Arabidopsis thaliana*	sp|Q9FWS9.1|
AtBLH4/SAW2	*Arabidopsis thaliana*	sp|Q94KL5.2|
AtBLH5	*Arabidopsis thaliana*	sp|Q8S897.2|
AtBLH6	*Arabidopsis thaliana*	sp|O65685.1|
AtBLH7/PNY	*Arabidopsis thaliana*	sp|Q9SIW1.1|
AtBLH8/PNF	*Arabidopsis thaliana*	sp|Q9SJJ3.1|
AtBLH9	*Arabidopsis thaliana*	sp|Q9LZM8.1|
AtBLH10	*Arabidopsis thaliana*	sp|Q9FXG8.1|
AtBLH11	*Arabidopsis thaliana*	sp|Q1PFD1.1|
StBEL5	*Solanum tuberosum*	AF406697
MDH1	*Malus domestica*	NP_001315679.1
GmBLH4	*Glycine max*	XP_006574714
WBLH2	*Triticum aestivum*	BAJ04687.1

TaqSH1/WBLH4	*Triticum aestivum*	BAJ04690.1
qSH1	*Oryza sativa Japonica Group*	XP_015641948.1
